# Posterior pericardiotomy to prevent new-onset atrial fibrillation after coronary artery bypass grafting: a systematic review and meta-analysis of 10 randomized controlled trials

**DOI:** 10.1186/s13019-021-01611-x

**Published:** 2021-08-14

**Authors:** Tao Xiong, Lei Pu, Yuan-Feng Ma, Yun-Long Zhu, Hua Li, Xu Cui, Ya-Xiong Li

**Affiliations:** 1 Department of Cardiac Surgery, Kunming Yan’an Hospital, Affiliated Hospital of Kunming Medical University, Kunming, 650000 Yunnan China; 2 Cardiovascular Surgery, Institution of Yunnan, Kunming, 650000 Yunnan China

**Keywords:** Posterior pericardiotomy, Postoperative atrial fibrillation, Coronary artery bypass grafting

## Abstract

**Background:**

Atrial fibrillation (AF) is associated with adverse events after cardiac surgery. Multiple studies have reported that posterior pericardiotomy (PP) may be effective for preventing AF after coronary artery bypass grafting (CABG), but some conflicting results have been reported and the quality of evidence from previous meta-analyses has been limited. The present study aimed to systematically evaluate the safety and efficacy of PP for preventing AF after CABG in adults.

**Methods:**

We conducted a quantitative meta-analysis of randomized controlled trials (RCTs) published before May 31, 2021. The primary outcome was AF after CABG under cardiopulmonary bypass. Secondary outcomes included early pericardial effusion, late pericardial effusion, pericardial tamponade, pleural effusion, length of hospital stay, length of intensive care unit (ICU) stay, pulmonary complications, intra-aortic balloon pump use, revision surgery for bleeding, and mortality.

**Results:**

Ten RCTs with 1829 patients (910 in the PP group and 919 in the control group) were included in the current meta-analysis. The incidence of AF was 10.3% (94/910) in the PP group and 25.7% (236/919) in the control group. A random-effects model indicated that incidence of AF after CABG significantly lower in the PP group than in the control group (risk ratio = 0.45, 95% confidence interval 0.29–0.64, *P* < 0.0001). PP also effectively reduced the post-CABG occurrence of early pericardial effusion (RR = 0.28, 95% CI 0.15–0.50; *P* < 0.05), late pericardial effusion (RR = 0.06, 95% CI 0.02–0.16; *P* < 0.05), and pericardial tamponade (RR = 0.08, 95% CI 0.02–0.33; *P* < 0.05) as well as the length of ICU stay (weighted mean difference [WMD] = 0.91,95% CI 0.57–1.24; *P* < 0.05), while increasing the occurrence pleural effusion (RR = 1.51, 95% CI 1.19–1.92; *P* < 0.05). No significant differences length of hospital stay (WMD =  − 0.45, 95% CI − 2.44 to 1.54, *P* = 0.66), pulmonary complications (RR = 0.99, 95% CI 0.71–1.39, *P* = 0.97), revision surgery for bleeding (RR = 0.84, 95% CI 0.43–1.63, *P* = 0.60), use of IABP (RR = 1, 95% CI 0.61–1.65, *P* = 1.0), or death (RR = 0.45, 95% CI 0.07–3.03, *P* = 0.41) were observed between the PP and control groups.

**Conclusions:**

PP may be a safe, effective, and economical method for preventing AF after CABG in adult patients.

**Supplementary Information:**

The online version contains supplementary material available at 10.1186/s13019-021-01611-x.

## Background

Postoperative atrial fibrillation (POAF) is one of the most common complications after cardiac surgery [1–3]. The true incidence of AF following cardiac surgery is controversial, ranging from 17 to 33% in the literature [4–6]. The incidence of POAF following coronary artery bypass grafting (CABG) surgery ranges between 20 and 40% [6, 7]. Although most POAF cases (> 90%) are cured within 4–6 weeks after surgery [8], the occurrence of POAF is related to increased risks of neurocognitive impairment [9], sepsis [10], embolic disease [11], congestive heart failure, and mortality [12], which in turn increases patients’ length of stay in the intensive care unit (ICU) [13] or the length of hospitalization, leading to greater costs [14, 15]. Therefore, a novel therapeutic approach for preventing POAF is a matter of high priority for reducing the duration of hospital stay, saving medical and material resources, reducing the occurrence of adverse events, and improving treatment outcomes. Over the past few decades, as interest in mediating risk factors and the use of anti-arrhythmic drugs have increased, more attention has been given to early-onset events related to the occurrence of POAF [16]. The pathophysiological mechanism of AF after CABG remains unclear though, even as studies have revealed that the main causes of POAF include inflammation, oxidative stress, autonomic dysfunction, and structure and electric remodeling [2]. Many drug interventions have been tested for their ability to reduce the incidence of POAF, but so far all have limitations and adverse effects [16] and are associated with significantly increased costs. Based on previous studies demonstrating a clear relationship between pericardial effusion and supra-ventricular arrhythmias, Mulay et al. [17] invented the technique of posterior pericardiotomy (PP). With this technique, at the end of surgery, a longitudinal incision is made parallel and posterior to the phrenic nerve, extending from the left inferior pulmonary vein to the diaphragm. Two chest drains are inserted, one in the left pleural cavity and the other in the anterior mediastinum, to fully drain the pericardial effusion, thereby reducing POAF. Multiple studies have confirmed that PP is a promising, economical, and effective technique for preventing POAF after cardiac surgery [17–20], because it can drain pericardial effusion to the left pleural cavity, which should reduce the risk of AF. However, conflicting results have been reported regarding the ability of PP to reduce the incidence of AF after CABG, with several studies finding that PP cannot reduce the incidence of AF after CABG [21–23] and two meta-analyses showing that PP can significantly reduce the incidence of AF after CABG [24, 25]. Notably, these previous meta-analyses contained insufficient research data and did not account for a lack of oral β-receptor blockade before surgery. In addition, the sample sizes of these studies were small, and they also did not analyze clinical heterogeneity. In order to evaluate more comprehensively the effectiveness of PP for preventing POAF, we conducted the present meta-analysis of a randomized trials to systematically evaluate the safety and effectiveness of PP for preventing AF after CABG in adults.

## Material and methods

We performed this meta-analysis in accordance with the Preferred Reporting Items for Systematic Reviews and Meta-analysis (PRISMA) statement [26]. Because all analyses were based on previous published studies, the requirements for ethical approval and patient consent were waived.

### Search strategy

The search strategy was based on the Cochrane System Review Manual [27]. Using the PICOS (Population, Intervention, Comparison, Results, and Study Design) standard search, we searched the PubMed, EMBASE, and Cochrane Library (including the Cochrane Central Registry of Controlled Trials) databases to retrieve all relevant articles published through May 2021. The free text and subject headings were combined. The search terms included “postpericardiotomy”, “pericardial fenestration”, “pericardial fenestration”, “CABG”, “coronary artery bypass grafting”, “heart surgery”, “cardiothoracic surgery”, “heart surgery”, “extracorporeal circulation”, and “CAB”. In order to maximize sensitivity, no language restrictions were applied. In addition, a manual search of the reference lists of the included studies was performed to identify other relevant publications. A more detailed description of the search strategy is provided in an Additional file 1: Appendix: search strategy.

### Eligibility criteria

The following inclusion criteria were used: (1) study design of randomized controlled trial (RCT); (2) study population of adult patients (≥ 18 years old) undergoing CABG surgery under cardiopulmonary bypass (CPB); (3) intervention based on random assignment to receive PP or conventional treatment (no PP); (4) comparison of PP group to control group (no PP); and (5) outcome measurement related to occurrence of AF.

### Exclusion criteria

The exclusion criteria were: treatment with surgery other than CABG, treatment without CPB, a non-randomized design, an animal study, and patients age < 18 years.

### Data extraction

All data were independently extracted by two researchers (M.Y.F and Z.Y.L) in duplicate. The following data were independently extracted using standardized and pilot data spreadsheets: first author, year of publication, type of surgery, study design, sample size, patient characteristics (age and percentage of males), intraoperative data, length of stay, length of ICU stay, AF, early pericardial effusion, late pericardial effusion, pleural effusion, pulmonary complications, intra-aortic ballon pump (IABP) use, pericardial tamponade, revision surgery for bleeding, death in the PP group, and number of individuals in the control group. The extracted data were checked by another investigator, and any inconsistencies were resolved through consensus and discussion. The primary outcome was the occurrence of AF.

### Risk of bias and strength of evidence assessments

The quality of evidence provided by each included study was evaluated using the recommended Cochrane bias risk assessment tool. The tool evaluates randomization (selection bias), allocation concealment (selection bias), blinding of research participants and personnel (performance bias), blinding of outcome assessment (detection bias), incomplete outcome data (attrition bias), selective reporting (reporting bias), and blinding of non-results information (other bias). Each study was assessed as having a low, unclear, or high risk of bias.

The Grades of Recommendations Assessment Development and Evaluation (GRADE) scale was used to assess the level and strength of evidence for recommendations, as follows: high quality: further research is unlikely to change our confidence in the estimation of effects; moderate quality: further research may affect our confidence in the effect estimate and may change the estimate; low quality: further research is likely to have an important impact on our confidence in the effect estimate and may change the estimate; and very low quality: we are very uncertain about this estimate.

### Trial sequential analysis (TSA)

To avoid the increased risk of Type I errors in this meta-analysis due to the scarcity of data and repeated cumulative data testing, TSA was used to determine whether evidence was reliable and conclusive. When the cumulative z-curve crosses the test sequence monitoring boundary or an invalid area, the level of evidence for intervention is sufficient and no further testing is required. If the z-curve does not cross any boundaries, there is not enough evidence to draw a conclusion, indicating that further research is still needed. In the current study, we used an alpha error of 0.05, a beta error of 0.20, a 20% reduction in the expected risk ratio (RR) of POAF, and the proportion of events from the control group in our meta-analysis to calculate the information sample size required for TSA.

### Outcome measures

We chose the main outcome to be the occurrence of POAF. Secondary outcomes included in-hospital or out-of-hospital mortality, pulmonary complications, early pericardial effusion, late pericardial effusion, pericardial tamponade, pleural effusion, length of stay in the hospital, length of stay in the ICU, use of IABP, and revision surgery for bleeding.

### Statistical analysis

For each basic hypothesis, the statistical significance level of the two-tailed test was 0.05. All statistical analyses were performed using Review Manager version 5.4 (The Cochrane Collaboration, Software Update, Oxford, UK) and STATA version 14 (Stata Corporation, College Station, TX, USA). The results are expressed with the Mantel–Haenszel RR and 95% confidence interval (CI) (using a fixed effects method, unless there was significant heterogeneity, in which case a random effects statistical model was used) [28]. We applied I^2^ and χ^2^ tests to test the heterogeneity between test results. Statistical heterogeneity was determined by evaluating the I^2^ value [29], according to the following ranges: 0–40%, possibly negligible heterogeneity; 30–60%, possibly moderate heterogeneity; 50–90%, possibly substantial heterogeneity; and 75–100%, potentially considerable heterogeneity [27]. All meta-analyses were performed using fixed or random effects models. Visual observation of Begg’s funnel chart along with Begg’s and Egger’s tests [30, 31] were used to assess publication bias.

## Results

### Literature search

**Fig. 1:**
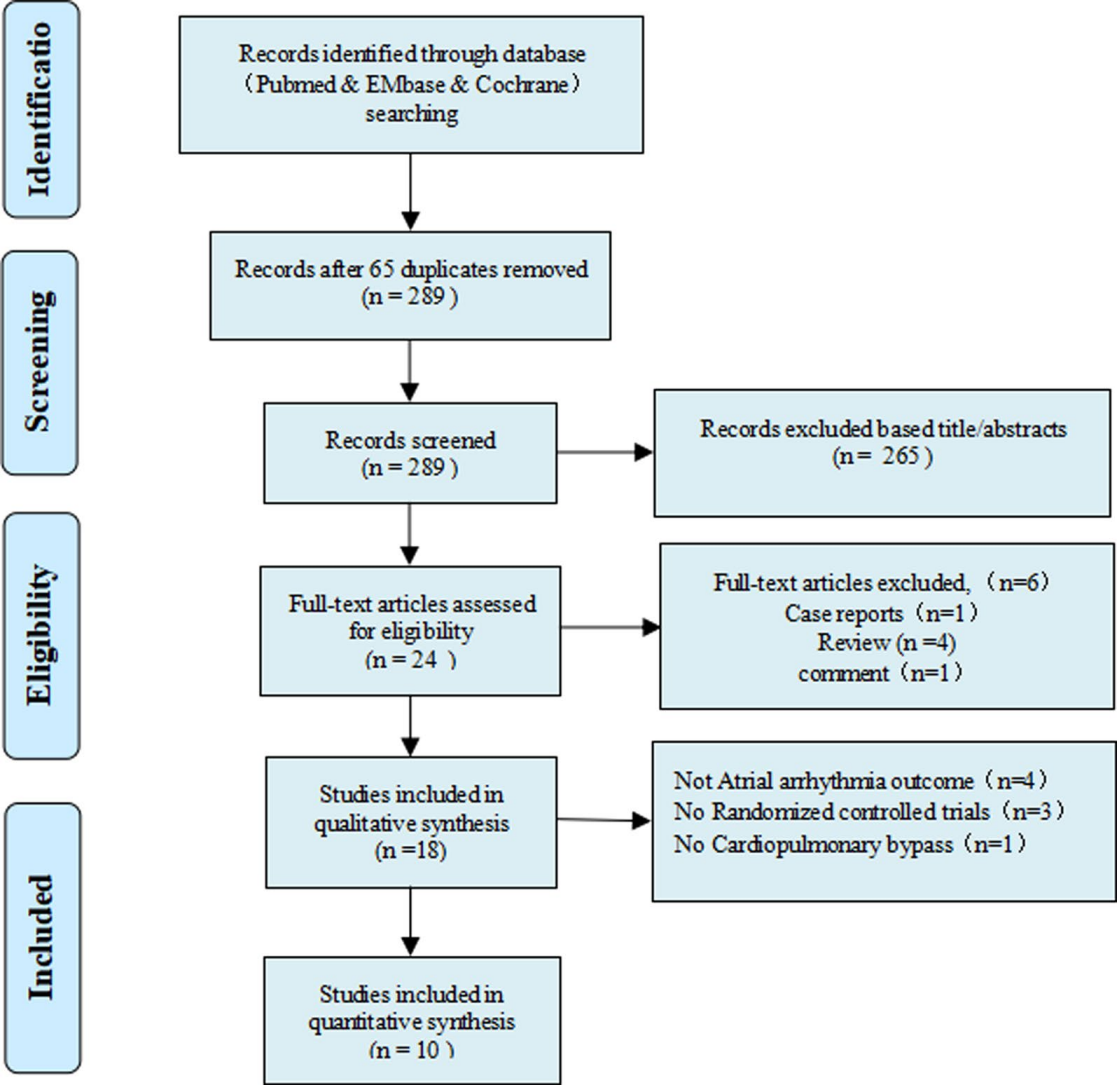
Flow chart of the selection process for studies included in the systematic review and meta-analysis

Figure 1 shows the flow chart of the literature research and study selection for this meta-analysis. The initial database search produced 289 related publications. After the exclusion of 65 duplicate studies and 265 studies deemed irrelevant based on titles and abstracts, the full text of remaining 24 studies was reviewed for detailed evaluation. Of these, 18 RCTs were included in the qualitative synthesis. Finally, a quantitative analysis of 10 studies (selected documents) was carried out, and the reasons for the exclusion of the eight studies are presented in Table 1.

**Table 1:** Reasons for exclusion of eight studies

Study	Reasons for exclusion
Mulay [17]	Not a randomized controlled trial
Goh [49]	Not a randomized controlled trial
Sperling [50]	Not a randomized controlled trial
Haddadzadeh [23]	Use of off-pump technology
Gulmen [51]	Outcome was not new onset of atrial fibrillation
Meza [52]	Outcome was not new onset of atrial fibrillation
Cakalagaoglu [53]	Outcome was not new onset of atrial fibrillation
Bakhshandeh [54]	Outcome was not new onset of atrial fibrillation

### Trial characteristics

**Fig. 2:**
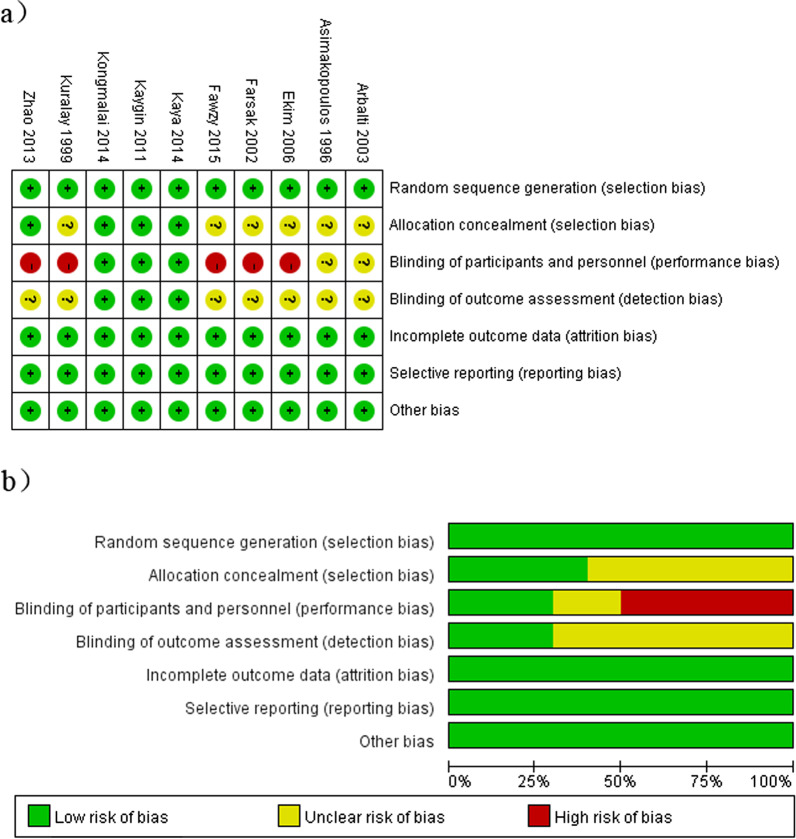
Quality assessments according risk of bias. **a** Risk of bias summary: judgments about each risk of bias item for each included study. **b** Risk of bias graph: judgments about each risk of bias item presented as percentages across all included studies

The characteristics of the 10 included RCTs and their participants are presented in Table 2, and the data reported by each included trial are described in Additional file 2: Table S1, and the actual mode of PP and the use of posterior pericardial drains are described in Additional file 2: Table S2. The included studies were published between 1995 and 2015, and the sample size for each ranged from 20 to 458, with a total of 1829 patients included in all 10 studies. All patients received CABG under CPB; the patients in four studies did not take β-blockers before the operation; and 6 studies were conducted in Turkey. Although the definition of AF varies, its main feature is that onset exceeding a certain period of time. Of the 10 included studies, all reported AF events as the main outcome [18–22, 29, 32–35], including early pericardial effusion in 9 studies [18–22, 29, 33–35], advanced pericardial effusion in 4 studies [18–20, 34], pulmonary complications in 6 studies [18–20, 22, 33, 34], pericardial tamponade in 7 studies [25, 37, 38, 40–43], pleural effusion in 6 studies [18–22, 29], length of hospital stay in 5 studies [18, 21, 22, 34, 35], use of IABP in 5 studies [24, 25, 36, 40, 43], length of ICU stay in 3 studies [21, 22, 29], revision surgery for bleeding in 3 studies [22, 33, 34], and mortality rate in 2 studies [32, 34]. All of the included studies were found to be of sufficient quality based on Cochrane bias risk assessment (Table 3 and Fig. 2).

**Table 2:** Main characteristics of randomized controlled trials included in the meta-analysis

Study ID	Area	Operation period	Study design	Surgery type	No. of patients (PP/control)	Age (years)		Male (%)		Cross-clamp time (min)		CPB time (min)	
						PP	Control	PP (%)	Control (%)	PP	Control	PP	Control
Kaya [34]	Turkey	2012.3–2013.1	RCT	CABG	63(30/33)	57 ± 10	59 ± 11	76.67	87.88	43 ± 16	46 ± 21	80 ± 26	86 ± 27
Kaygin [33]	Turkey	2009.8–2011.2	RCT	CABG	425(213/212)	59 ± 11	59 ± 11	50.23	49.53	> 50	> 50	> 80	> 80
Fawzy [35]	Egypt	2010.6–2012.5	RCT	CABG	200(100/100)	54 ± 9	56 ± 10	64.00	68.00	55 ± 21	59 ± 17	89 ± 29	87 ± 23
Zhao [29]	China	2012.1–2013.1	RCT	CABG	458(228/230)	54 ± 16	56 ± 18	60.53	54.35	67 ± 29	62 ± 23	110 ± 46	103 ± 51
Kuralay [18]	Turkey	1996.6–1997.6	RCT	CABG	200(100/100)	57 ± 12	61 ± 8	77	73	36 ± 12	43 ± 9	48 ± 5	51 ± 4
Asimakopoulos [32]	UK	NR	RCT	CABG	100(50/50)	60 ± 9	60 ± 9	NR	NR	35	33	66	62
Ekim [20]	Turkey	2003.10–2005.7	RCT	CABG	100(50/50)	59 ± 9	60 ± 3	66	64	63 ± 19	62 ± 12	89 ± 21	87 ± 26
Farsak [19]	Turkey	2000.4–2001.10	RCT	CABG	150(75/75)	64 ± 9	63 ± 5	36	32	35 ± 11	40 ± 9	58 ± 6	61 ± 9
Kongmalai [22]	Thailand	2013.8–2013.12	RCT	CABG	20(10/10)	65 ± 13	59 ± 5	50.00	50.00	84 ± 38	107 ± 39	128 ± 49	152 ± 45
Arbatli [21]	Turkey	2000.5–2000.12	RCT	CABG	113(54/59)	62 ± 8	60 ± 9	83.33	74.58	58 ± 17	60 ± 19	117 ± 32	112 ± 35

CABG, coronary artery bypass grafting; POAF, postoperative atrial fibrillation; ICU, intensive care unit; RCT, randomized controlled trial; NR, not reported; CPB, cardiopulmonary bypass; IABP, intra-aortic balloon pump

**Table 3:** Methodological quality of included studies

Study ID	Random sequence generation (selection bias)	Allocation concealment (selection bias)	Blinding of participants and personnel (performance bias)
Kaya [34]	Random number table	Described blinding	All patients provided informed consent and were treated blindly
Kaygin [33]	Described randomization	Described blinding	All patients provided informed consent and were treated blindly
Fawzy [35]	Described randomization	Not described	All patients provided informed consent
Zhao [29]	Random number table	Opaque envelope	All patients provided informed consent
Kuralay [18]	Random number table	Not described	All patients provided informed consent
Asimakopoulos [32]	Described randomization	Not described	Not described
Ekim [20]	Described randomization	Not described	All patients provided informed consent
Farsak [19]	Random number table	Not described	All patients provided informed consent
Kongmalai [22]	Described randomization	Opaque envelope	Patients provided informed written consent individually
Arbatli [21]	Described randomization	Not described	Not described

Blinding of outcome assessment (detection bias)	Incomplete outcome data (attrition bias)	Selective reporting (reporting bias)	Other bias
Described blinding	Complete	No	No
Described blinding	Complete	No	No
Not described	Complete	No	No
Not described	Complete	No	No
Not described	Complete	No	No
Not described	Complete	No	No
Not described	Complete	No	No
Not described	Complete	No	No
Researchers analyzed data blinded	Complete	No	No
Not described	Complete	No	No

### PP and POAF

**Fig. 3:**
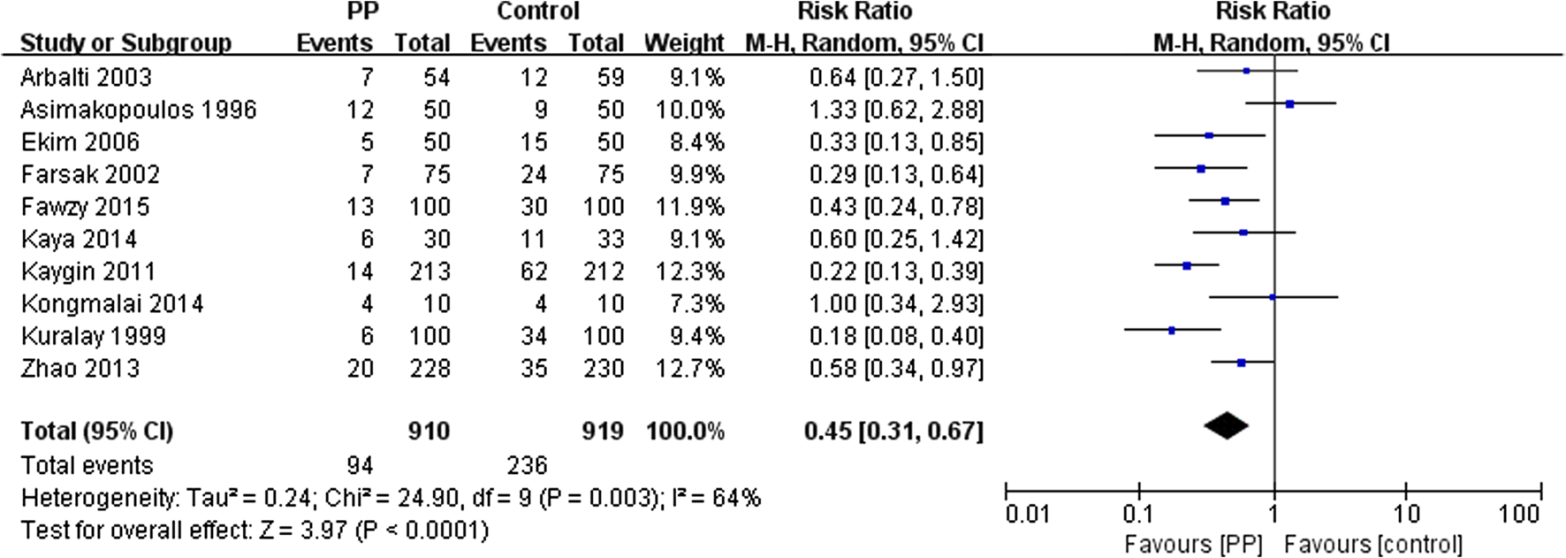
Pooled estimates from RCTs evaluating effects of PP on the incidence of AF after CABG surgery with a random-effects model. PP, posterior pericardiotomy; CI, confidence interval

**Fig. 4:**
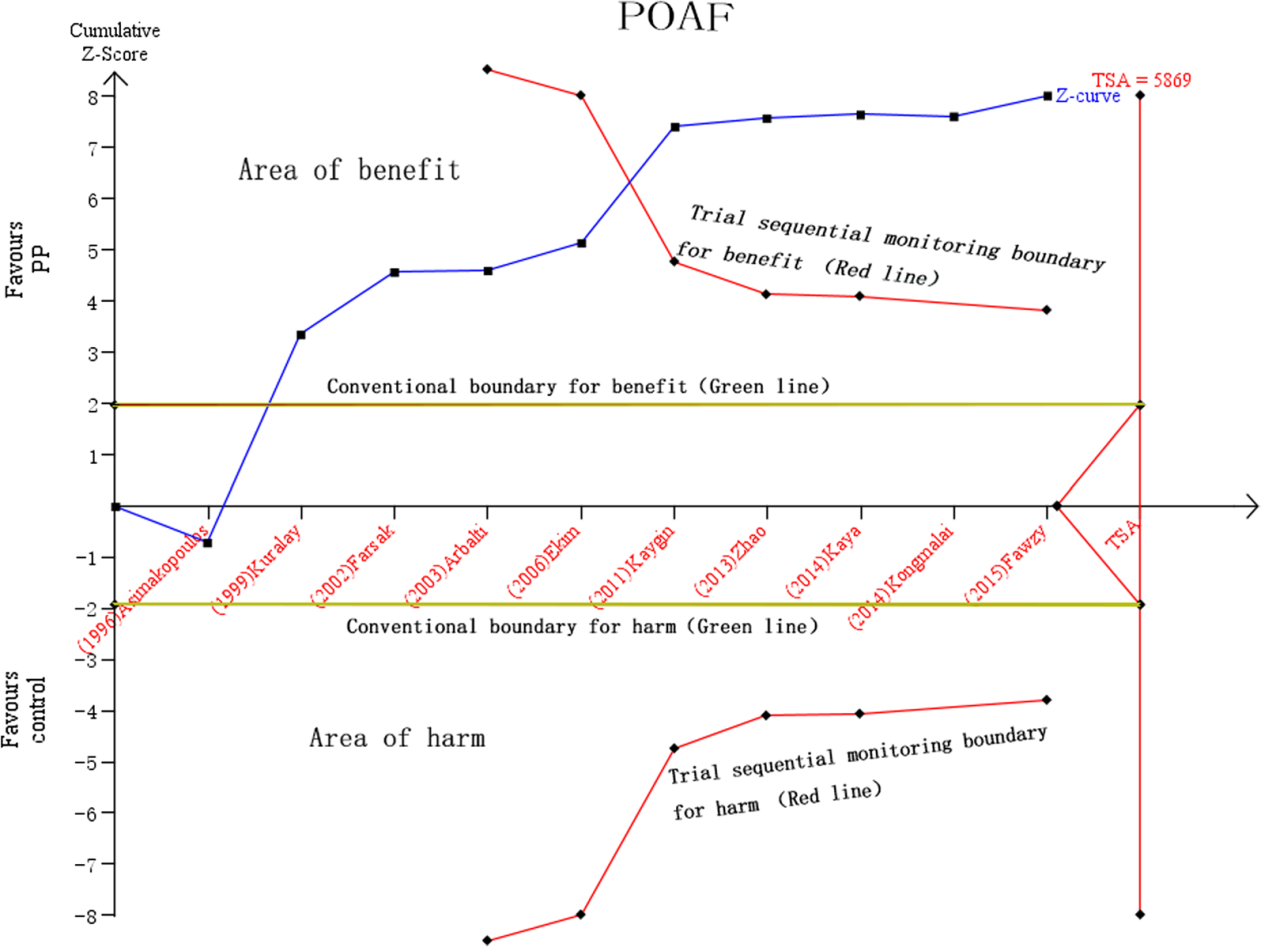
Trial sequential analysis of 10 RCTs (black square icons) illustrating that the cumulative z-curve crossed both the conventional boundary for benefit and the trial sequential monitoring boundary for benefit and entered the area of benefit, establishing sufficient and conclusive evidence and suggesting further trials are not needed. A diversity adjusted required information size of 5869 patients was calculated using an alpha error of 0.05, a beta error of 0.20 (power 80%), an anticipated RR reduction of 20% in AF, and a control event proportion of 25.3%, as calculated from the control group in our meta-analysis

A total of 1829 participants were included in the present analysis (910 in the PP group and 919 in the control group). The cumulative incidence of AF was 10.3% in the PP group and 25.7% in the control group [18–20, 29, 32–35]. Fixed effects model analysis (I^2^ = 64%, Q-test *P* = 0.003, and effect size RR = 0.40 [95% CI 0.32–0.50, *P* < 0.05) indicated heterogeneity among the selected studies. On sensitivity analysis, further exclusion of any single study did not substantially change the overall combined RR, which ranged from 0.37 (95% CI 0.29–0.48) to 0.47 (95% CI 0.37–0.59). The random effects model combined with RR showed that PP significantly reduced the incidence of AF after CABG (RR 0.45, 95% CI 0.31–0.67; *P* < 0.05; Fig. 3), and moderate heterogeneity was observed among studies (*P* = 0.003, I^2^ = 64%). As shown in Fig. 4, the z-curve on TSA entered the benefit area and crossed the conventional benefit boundary as well as the experimental sequential monitoring benefit boundary, indicating that the evidence was sufficient and conclusive, requiring no further research.

### Sensitivity and subgroup analyses

We next tested the source of heterogeneity via a sensitivity analysis. Upon exclusion of the study with the smallest sample size [22], the fixed-effects model showed significant heterogeneity (I^2^ = 64%, *P* = 0.004; Additional file 2: Figure S1). Thus, the random-effects model was used and provided results similar to the overall results (I^2^ = 64%, *P* = 0.004; RR = 0.45; 95% CI 0.31–0.67, *P* < 0.0001), which were statistically significant and provided substantial evidence of heterogeneity. In addition, this analysis may be affected by the use of β-blockers. Four studies in which patients did not take β-blockers before surgery were analyzed and no significant heterogeneity was observed (I^2^ = 35%, Q-test *P* = 0.2; Additional file 2: Figure S2). Pooled analysis of these four studies using the fixed-effect model showed that PP had a good effect on reducing the incidence of AF after CABG without being affected by β-blockers (RR = 0.30, 95% CI 0.20–0.45; *P* < 0.05), with statistical significance [18–21]. As mentioned above, 60% of the included studies were conducted in Turkish populations. Therefore, we examined the clinical heterogeneity caused by geographic area. When we pooled and analyzed the Turkish studies only, we found no significant heterogeneity (I^2^ = 40%, *P* = 0.14; Additional file 2: Figure S3), and using the fixed-effects model, these studies showed that PP had a good effect on reducing the incidence of AF after CABG without being affected by clinical heterogeneity (RR = 0.29, 95% CI 0.21–0.39; *P* < 0.05) [18–21, 33, 34].

### Secondary outcomes

Compared with the control treatment, PP effectively reduced the post-CABG occurrence of early pericardial effusion (RR = 0.28, 95% CI 0.15–0.50; *P* < 0.05; Additional file 2: Figure S4), late pericardial effusion (RR = 0.06, 95% CI 0.02–0.16; *P* < 0.05; Additional file 2: Figure S5), and pericardial tamponade (RR = 0.08, 95% CI 0.02–0.33; *P* < 0.05; Additional file 2: Figure S6) as well as the length of ICU stay (weighted mean difference [WMD] = 0.91,95% CI 0.57–1.24; *P* < 0.05; Additional file 2: Figure S7), while increasing the occurrence of pleural effusion (RR = 1.51, 95% CI 1.19–1.92; *P* < 0.05; Additional file 2: Figure S8). As demonstrated by the data in Table 4, no significant differences were observed between the PP and control groups in terms of length of hospital stay (WMD =  − 0.45, 95% CI − 2.44 to 1.54, *P* = 0.66; Additional file 2: Figure S9); incidence of pulmonary complications (RR = 0.99, 95% CI 0.71–1.39, *P* = 0.97; Additional file 2: Figure S10), revision surgery for bleeding (RR = 0.84, 95% CI 0.43–1.63, *P* = 0.60; Additional file 2: Figure S11), use of IABP (RR = 1, 95% CI 0.61–1.65, *P* = 1.0; Additional file 2: Figure S12), or death (RR = 0.45, 95% CI 0.07–3.03, *P* = 0.41; Additional file 2: Figure S13).

**Table 4:** Subgroup analysis of adverse events

Adverse events after surgery	Results of subgroup pool analysis	*P* value
Early pericardial effusion	RR: 0.28; 95% CI 0.15–0.50	< 0.0001
Late pericardial effusion	RR: 0.06; 95% CI 0.02–0.16	< 0.00001
Pericardial tamponade	RR: 0.08; 95% CI 0.02–0.33	0.0005
Length of stay in intensive care unit (ICU)	MD: 0.91; 95% CI 0.57–1.24	< 0.00001
Pleural effusion	RR: 1.51; 95% CI 1.19–1.92	0.0007
Length of hospitalization	MD: − 0.45; 95% CI − 2.44–1.54	0.66
Pulmonary complications	RR: 0.99; 95% CI 0.71–1.39	0.97
Revision for bleeding	RR: 0.84; 95% CI 0.43–1.63	0.60
Intra-aortic balloon pump (IABP) usage	RR: 1.00; 95% CI 0.61–1.65	1.00
Death	RR: 0.45; 95% CI 0.07–3.03	0.41

RR, risk ratio; MD, mean difference; CI, confidence interval

### GRADE assessment and publication bias

**Fig. 5:**
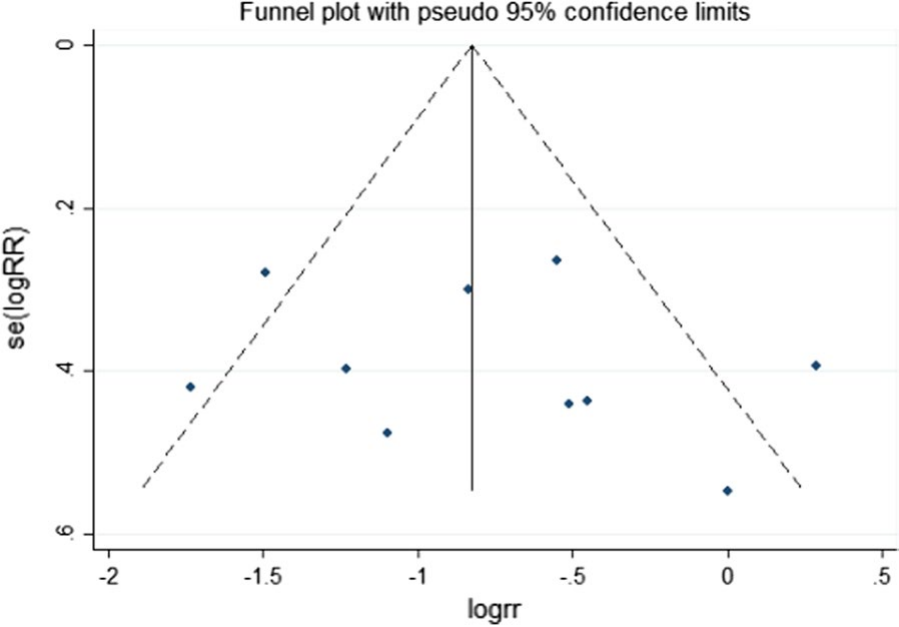
Tests for publication bias among the 10 included studies on the effect of PP on AF after CABG

**Fig. 6:**
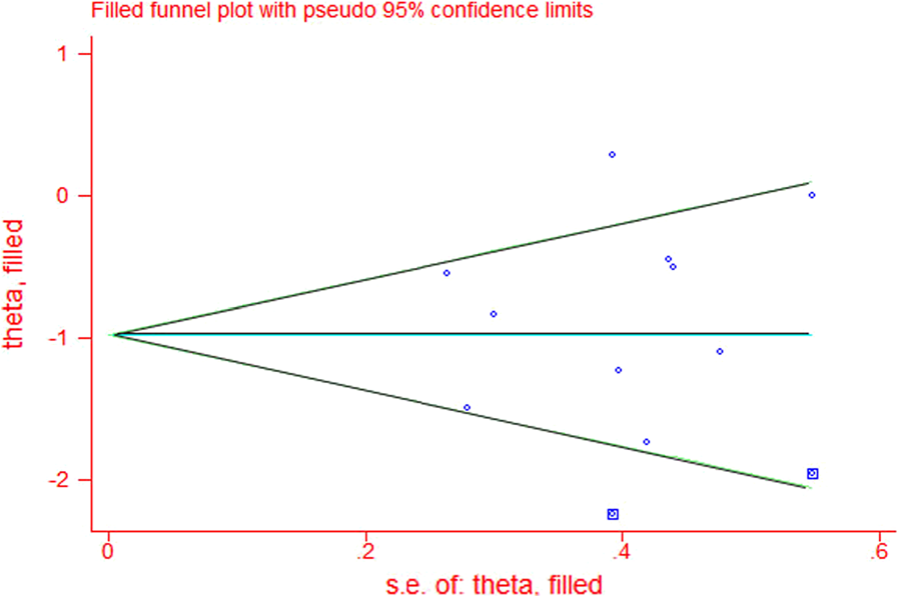
Improvement of the stability of the results through the trim and fill method

The GRADE rating results are shown in Table 5. According to the GRADE system, the strength of evidence was high for pericardial tamponade, pleural effusion, and early pericardial effusion; moderate for AF, ICU stay, and late pericardial effusion; low for pulmonary complications; and extremely low for revision surgery for bleeding, IABP use, hospital stay, and death. Visual inspection of the funnel chart revealed asymmetry, indicating the possibility of moderate publication bias (Fig. 5). However, further bias testing via the Egger test and Begg test resulted in P values of 0.532 and 0.721, respectively. Thus, there was no statistical evidence of publication bias. The funnel chart for this study is therefore ambiguous, as the *P* values from both tests were > 0.1. We also considered that the study was moderately heterogeneous. Therefore, the stability of this study was analyzed by the trim and fill method. The results in Fig. 6 show that two more studies are needed to improve the stability of the results. This finding shows that there was a certain publication bias. Possibly due to the insufficient volume of the literature and clinical heterogeneity, the effectiveness of the test was insufficient.

**Table 5:**
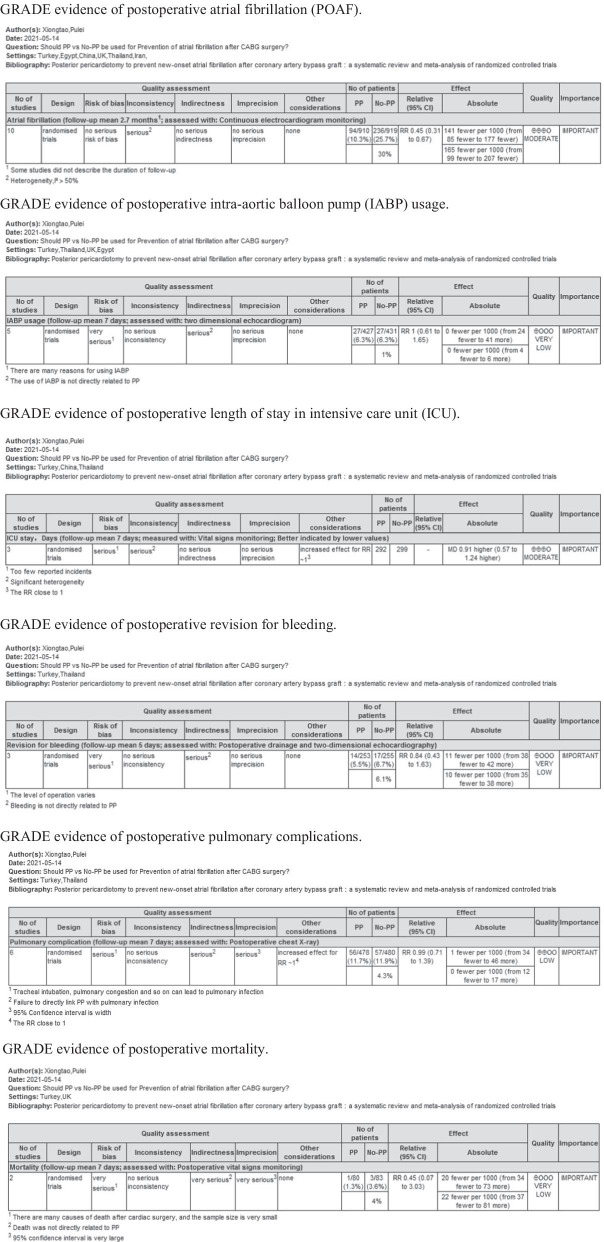

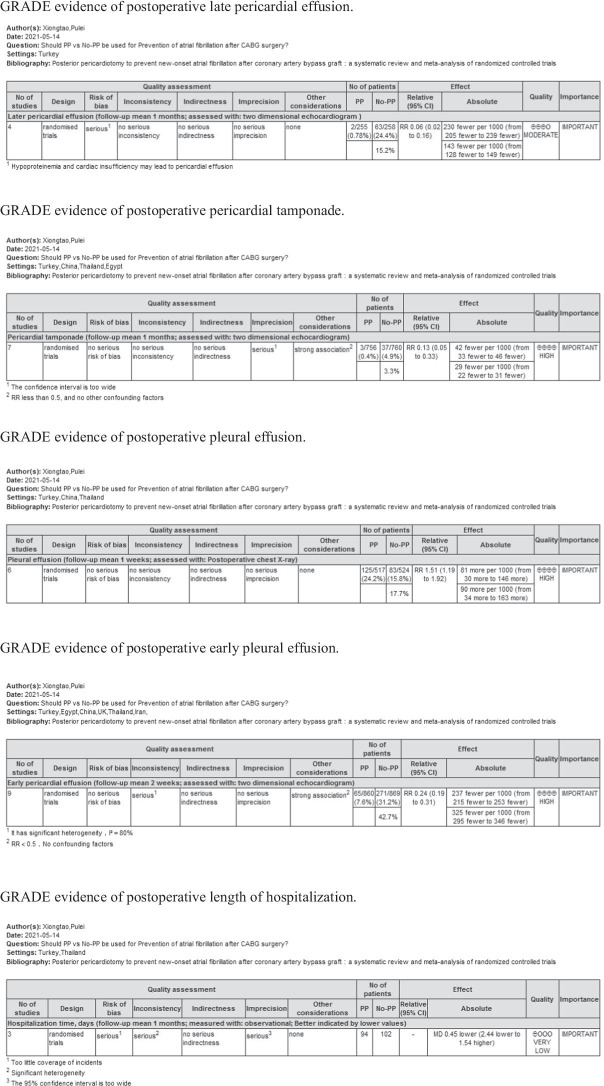
GRADE evidence profile

## Discussion

In this systematic review and meta-analysis of 10 prospective RCTs on the effectiveness of PP for preventing AF after CABG in adult patients, the comprehensive results of the random effects model showed that PP had a good effect in preventing AF after CABG. Although some RCTs have reported conflicting results [21–23], the findings of the present study are similar to the results of previous meta-analyses [24, 25]. Compared with the previous meta-analyses, the present study offers the advantages of an effect size based on RR, the inclusion of all RCT trials in which CABG was assisted by CPB, and the inclusion of two additional RCTs, which further improved upon the quality of the study. Tests for heterogeneity returned an I^2^ = 64% and Q-test *P* = 0.003, suggesting that there was heterogeneity between the studies, which may be due to clinical heterogeneity or variation in the use of β-blockers before surgery [18–21]. Although our results are generally consistent with the main results of the previous meta-analyses [24, 25], the present study has expanded this line of research in several important aspects. The present study applied TSA for power analysis, which verified that the evidence was sufficient and conclusive. In addition, the evidence for the use of PP for CABG patients was graded and evaluated. The quality of the evidence for the prevention of POAF was high, and the previous meta-analyses lacked this evaluation.

While the previous meta-analyses reported that PP significantly reduced the incidence of AF after cardiac surgery [24, 25], those systematic reviews did not control for the use of β-blockers or for ethnic differences in comparing the PP and control groups. Moreover, previous research has indicated that non-dihydropyridine calcium channel blockers [36], magnesium [37], vitamins [38], polyunsaturated fatty acids [39], corticosteroids [40], colchicine [41], Reynolds Triazine [42], glucocorticoids [43], antiarrhythmic drugs [44], and statins [45] are all related to the incidence of AF after cardiac surgery. Based on their good effects of β-blockers, their use is considered a Class I recommendation in the guidelines of the American College of Cardiology/American Heart Association, European College of Cardiology, and American Thoracic Surgery Association [8, 46, 47]. Therefore, our analysis multiple studies [18–21] in which patients did not take β-blockers before surgery showed that PP reduced the incidence of AF after CABG (RR = 0.30, 95% CI 0.20–0.45, *P* < 0.00001; I^2^ = 35%, *P* = 0.20), which further clarified the efficacy of PP for preventing AF after CABG. At the same time, in this study, because the included studies were all RCTs, clinical heterogeneity was the main source of statistical heterogeneity, with studies in Turkey accounting for 60% of all studies. To further exclude clinical heterogeneity, we performed a meta-analysis of the six prospective RCTs from Turkey, and the results again showed that PP reduced the incidence of AF after CABG (RR = 0.32, 95% CI 0.23–0.45, *P* < 0.00001; I^2^ = 35%, *P* = 0.19). While these findings may indicate that PP can improve the incidence of AF after CABG, we cannot rule out the effects of potential bias in the included studies.

Our analysis of the effects of PP on early pericardial effusion, late pericardial effusion, pleural effusion, pulmonary complications, length of hospital stay, length of ICU stay, IABP use, and mortality produced varying results. With regard to postoperative pericardial effusion, our study showed that the incidence of early pericardial effusion (RR = 0.28, 95% CI 0.15–0.50; *P* < 0.05) and the incidence of advanced pericardial effusion (RR = 0.06, 95% CI 0.02–0.16; *P* < 0.05) were significantly lower in the PP group than in the control group, indicating that PP can significantly reduce the incidence of pericardial effusion in patients after CABG. Previous research has shown that the incidence of supraventricular arrhythmias is higher in patients with pericardial effusion than in those without pericardial effusion [48], and the earliest studies showed that PP can reduce the incidence of supraventricular arrhythmias while reducing pericardial effusion after CABG [17]. Therefore, pericardial effusion may be related to the occurrence of AF after CABG.

The present meta-analysis showed that PP effectively reduced the incidence of postoperative cardiac tamponade compared with that in the control group (RR = 0.08, 95% CI 0.02–0.33; *P* < 0.05). In addition, the incidence of pleural effusion in the PP group was significantly higher than that in the control group (RR = 1.51, 95% CI 1.19–1.92; *P* < 0.05), indicating that through the PP process, fluid can be discharged freely into the left thoracic cavity, thereby significantly reducing the incidence of pericardial tamponade [18–20, 29, 32–35]. Importantly, the incidence of pulmonary complications did not differ significantly between the PP group and the control group (RR = 0.99, 95% CI 0.71–1.39, *P* = 0.97). Therefore, the present study indicates that PP provides an effective method for chest drainage to reduce the occurrence of cardiac tamponade without increasing the risk of pulmonary complications. More trials are needed to confirm this finding.

Our analysis of the postoperative ICU stay showed that PP shortened the ICU stay compared with that in the control group (WMD = 0.91, 95% CI 0.57–1.24; *P* < 0.05). Because the duration of treatment in the ICU is related to total hospitalization expenses [33], PP may be a safe, effective, and economical method for preventing AF after CABG surgery that reduces patients’ medical expenses and saves hospital resources. A large number of similar trials is still needed to confirm this finding. Our analysis showed no differences in the postoperative hospital stay (WMD =  − 0.45, 95% CI − 2.44 to 1.54, *P* = 0.66), IABP use (RR = 1, 95% CI 0.61–1.65, *P* = 1.0), need for revision surgery for bleeding (RR = 0.84, 95% CI 0.43–1.63, *P* = 0.60), or mortality (RR = 0.45, 95% CI 0.07–3.03, *P* = 0.41) between the PP and control groups. Although we found no correlation between PP and these outcomes, these events were very infrequent, which precluded a comprehensive safety assessment of these results. While these results may indicate that the PP procedure did not affect hospital stay, lung complications, revision surgery for bleeding, use of IABP, and mortality, this was an observational analysis that could produce misleading results. Therefore, these results should be interpreted with caution.

### Limitations of this study

Our systematic review has certain limitations. First, although our results are consistent with those of previous systematic reviews, only 3 of the 10 included RCTs provided high-quality evidence [22, 34], and the inclusion criteria for our analysis did not allow for the inclusion of studies comparing other drugs to surgery. Also, the included studies did not strictly control for the effects of preoperative drugs (β-blockers, CCB, ACEIs, etc.) on postoperative POAF recurrence. Patients did not take β-blockers before surgery in only four studies, and thus, this analysis still cannot provide enough effective evidence to ensure the validity of the results. Second, although no differences in pulmonary complications, IABP use, revision surgery for bleeding, and mortality were detected between the PP and control groups of the 10 studies, these events were infrequently reported. Thus, these results may be misleading.

In addition, there was moderate heterogeneity among the included studies, which can be attributed to differences in patient characteristics, study design, and the definition of new POAF, resulting in unstable analysis results. One of the 10 studies [22] had a small sample size of only 20 patients, which may potentially underestimate the incidence of AF after CABG. Finally, six included studies [18–21, 33, 34] were conducted in one geographic area (Turkey), creating the potential for bias.

## Conclusion

In summary, in this systematic review and meta-analysis, PP showed good effects for preventing pericardial effusion, pericardial tamponade, and new-onset AF after CABG in adults with few related complications. These findings indicate that PP is a simple and safe surgical method without obvious complications. However, the quality of the included studies was limited, and the mortality and complication data were insufficient. More high-quality RCTs are still needed to assess the safety of PP for preventing AF after CABG.

## Supplementary Information

**Additional file 1**: Appendix: Search strategy.

**Additional file 2: Figure S1.** Pooled analysis for the comparison of the risk for postoperative atrial fibrillation (POAF) after removal of the study with the smallest sample size. **Figure S2.** Pooled analysis for the comparison of the risk for postoperative atrial fibrillation (POAF) without preoperative oral β-blockers. **Figure S3.** Pooled analysis for the comparison of the risk for postoperative atrial fibrillation (POAF) in Turkey. **Figure S4.** Pooled analysis for the comparison of the risk for postoperative early pericardial effusion. **Figure S5.** Pooled analysis for the comparison of the risk for postoperative late pericardial effusion. **Figure S6.** Pooled analysis for the comparison of the risk for postoperative pericardial tamponade. **Figure S7.** Pooled analysis for the comparison of the risk for postoperative length of stay in intensive care unit (ICU). **Figure S8.** Pooled analysis for the comparison of the risk for postoperative pleural effusion. **Figure S9.** Pooled analysis for the comparison of the risk for postoperative length of hospitalization. **Figure S10.** Pooled analysis for the comparison of the risk for postoperative pulmonary complications. **Figure S11.** Pooled analysis for the comparison of the risk for postoperative revision for bleeding. **Figure S12.** Pooled analysis for the comparison of the risk for postoperative intra-aortic balloon pump (IABP) usage. **Figure S13.** Pooled analysis for the comparison of the risk for postoperative death. **Table S1.** Main postoperative data from random controlled trials included in the meta-analysis. **Table S2.** Actual mode of PP and use of posterior pericardial drains.

## Data Availability

The datasets used and/or analysed during the current study are available from the corresponding author on reasonable request.

## Abbreviations

POAF: Postoperative atrial fibrillation
AF: Atrial fibrillation
CABG: Coronary artery bypass grafting
ICU: Intensive care unit
PP: Posterior pericardiotomy
PRISMA: Systematic Reviews and Meta-analysis
PICOS: Population: Intervention: Comparison: Results: and Study Design
RCT: Randomized controlled trial
CPB: Cardiopulmonary bypass
IABP: Intra-aortic balloon pump
GRADE: The Grades of Recommendations Assessment Development and Evaluation
TSA: Trial sequential analysis
RR: Risk ratio
NR: Not reported

## References

[1] Burgess DC, Kilborn MJ, Keech AC (2006). Interventions for prevention of post-operative atrial fibrillation and its complications after cardiac surgery: a meta-analysis. Eur Heart J.

[2] Echahidi N, Pibarot P, O'Hara G, Mathieu P (2008). Mechanisms, prevention, and treatment of atrial fibrillation after cardiac surgery. J Am Coll Cardiol.

[3] Raiten J, Patel PA, Gutsche J (2015). Management of postoperative atrial fibrillation in cardiac surgery patients. Semin Cardiothorac Vasc Anesth.

[4] Chung MK (2000). Cardiac surgery: postoperative arrhythmias. Crit Care Med.

[5] Hakala T, Hedman A (2003). Predicting the risk of atrial fibrillation after coronary artery bypass surgery. Scand Cardiovasc J.

[6] Mathew JP, Fontes ML, Tudor IC, Ramsay J, Duke P, Mazer CD, Barash PG, Hsu PH, Mangano DT (2004). A multicenter risk index for atrial fibrillation after cardiac surgery. JAMA.

[7] Haghjoo M (2012). Pharmacological and nonpharmacological prevention of atrial fibrillation after coronary artery bypass surgery. J Tehran Heart Cent.

[8] Frendl G, Sodickson AC, Chung MK, Waldo AL, Gersh BJ, Tisdale JE, Calkins H, Aranki S, Kaneko T, Cassivi S (2014). 2014 AATS guidelines for the prevention and management of perioperative atrial fibrillation and flutter for thoracic surgical procedures. J Thorac Cardiovasc Surg.

[9] Selnes OA, Gottesman RF, Grega MA, Baumgartner WA, Zeger SL, McKhann GM (2012). Cognitive and neurologic outcomes after coronary-artery bypass surgery. N Engl J Med.

[10] Kalavrouziotis D, Buth KJ, Ali IS (2007). The impact of new-onset atrial fibrillation on in-hospital mortality following cardiac surgery. Chest.

[11] Al-Shaar L, Schwann TA, Kabour A, Habib RH (2014). Increased late mortality after coronary artery bypass surgery complicated by isolated new-onset atrial fibrillation: a comprehensive propensity-matched analysis. J Thorac Cardiovasc Surg.

[12] Yadava M, Hughey AB, Crawford TC (2014). Postoperative atrial fibrillation: incidence, mechanisms, and clinical correlates. Cardiol Clin.

[13] Mathew JP, Parks R, Savino JS, Friedman AS, Koch C, Mangano DT, Browner WS (1996). Atrial fibrillation following coronary artery bypass graft surgery: predictors, outcomes, and resource utilization. MultiCenter Study of Perioperative Ischemia Research Group. Jama.

[14] Andrews TC, Reimold SC, Berlin JA, Antman EM (1991). Prevention of supraventricular arrhythmias after coronary artery bypass surgery: a meta-analysis of randomized control trials. Circulation.

[15] Omae T, Kanmura Y (2012). Management of postoperative atrial fibrillation. J Anesth.

[16] Koniari I, Apostolakis E, Rogkakou C, Baikoussis NG, Dougenis D (2010). Pharmacologic prophylaxis for atrial fibrillation following cardiac surgery: a systematic review. J Cardiothorac Surg.

[17] Mulay A, Kirk AJ, Angelini GD, Wisheart JD, Hutter JA (1995). Posterior pericardiotomy reduces the incidence of supra-ventricular arrhythmias following coronary artery bypass surgery. Eur J Cardiothorac Surg.

[18] Kuralay E, Ozal E, Demirkili U, Tatar H (1999). Effect of posterior pericardiotomy on postoperative supraventricular arrhythmias and late pericardial effusion (posterior pericardiotomy). J Thorac Cardiovasc Surg.

[19] Farsak B, Günaydin S, Tokmakoğlu H, Kandemir O, Yorgancioğlu C, Zorlutuna Y (2002). Posterior pericardiotomy reduces the incidence of supra-ventricular arrhythmias and pericardial effusion after coronary artery bypass grafting. Eur J Cardiothorac Surg.

[20] Ekim H, Kutay V, Hazar A, Akbayrak H, Başel H, Tuncer M (2006). Effects of posterior pericardiotomy on the incidence of pericardial effusion and atrial fibrillation after coronary revascularization. Med Sci Monit.

[21] Arbatli H, Demirsoy E, Aytekin S, Rizaoglu E, Unal M, Yagan N, Sonmez B (2003). The role of posterior pericardiotomy on the incidence of atrial fibrillation after coronary revascularization. J Cardiovasc Surg (Torino).

[22] Kongmalai P, Karunasumetta C, Kuptarnond C, Prathanee S, Taksinachanekij S, Intanoo W, Wongbuddha C, Senthong V (2014). The posterior pericardiotomy. Does it reduce the incidence of postoperative atrial fibrillation after coronary artery bypass grafting?. J Med Assoc Thai.

[23] Haddadzadeh M, Motavaselian M, Rahimianfar AA, Forouzannia SK, Emami M, Barzegar K (2015). The effect of posterior pericardiotomy on pericardial effusion and atrial fibrillation after off-pump coronary artery bypass graft. Acta Med Iran.

[24] Biancari F, Mahar MA (2010). Meta-analysis of randomized trials on the efficacy of posterior pericardiotomy in preventing atrial fibrillation after coronary artery bypass surgery. J Thorac Cardiovasc Surg.

[25] Hu XL, Chen Y, Zhou ZD, Ying J, Hu YH, Xu GH (2016). Posterior pericardiotomy for the prevention of atrial fibrillation after coronary artery bypass grafting: a meta-analysis of randomized controlled trials. Int J Cardiol.

[26] Moher D, Liberati A, Tetzlaff J, Altman DG (2009). Preferred reporting items for systematic reviews and meta-analyses: the PRISMA statement. Ann Intern Med.

[27] Higgins J. Cochrane handbook for systematic reviews of interventions. Version 5.1. 0 [updated March 2011]. The Cochrane Collaboration. 2011. https://www.cochrane-handbook.org.

[28] DerSimonian R, Kacker R (2007). Random-effects model for meta-analysis of clinical trials: an update. Contemp Clin Trials.

[29] Zhao J, Cheng Z, Quan X, Zhao Z (2014). Does posterior pericardial window technique prevent pericardial tamponade after cardiac surgery?. J Int Med Res.

[30] Egger M, Davey Smith G, Schneider M, Minder C (1997). Bias in meta-analysis detected by a simple, graphical test. BMJ.

[31] Begg CB, Mazumdar M (1994). Operating characteristics of a rank correlation test for publication bias. Biometrics.

[32] Asimakopoulos G, Della Santa R, Taggart DP (1997). Effects of posterior pericardiotomy on the incidence of atrial fibrillation and chest drainage after coronary revascularization: a prospective randomized trial. J Thorac Cardiovasc Surg.

[33] Kaygin MA, Dag O, Güneş M, Senocak M, Limandal HK, Aslan U, Erkut B (2011). Posterior pericardiotomy reduces the incidence of atrial fibrillation, pericardial effusion, and length of stay in hospital after coronary artery bypasses surgery. Tohoku J Exp Med.

[34] Kaya M, İyigün T, Yazıcı P, Melek Y, Göde S, Güler S, Karaçalılar M, Satılmışoğlu MH, Erek E (2014). The effects of posterior pericardiotomy on pericardial effusion, tamponade, and atrial fibrillation after coronary artery surgery. Kardiochir Torakochirurgia Pol.

[35] Fawzy H, Elatafy E, Elkassas M, Elsarawy E, Morsy A, Fawzy A (2015). Can posterior pericardiotomy reduce the incidence of postoperative atrial fibrillation after coronary artery bypass grafting?. Interact Cardiovasc Thorac Surg.

[36] Wijeysundera DN, Beattie WS, Rao V, Karski J (2003). Calcium antagonists reduce cardiovascular complications after cardiac surgery: a meta-analysis. J Am Coll Cardiol.

[37] Fairley JL, Zhang L, Glassford NJ, Bellomo R (2017). Magnesium status and magnesium therapy in cardiac surgery: a systematic review and meta-analysis focusing on arrhythmia prevention. J Crit Care.

[38] Dehghani MR, Majidi N, Rahmani A, Asgari B, Rezaei Y (2014). Effect of oral vitamin C on atrial fibrillation development after isolated coronary artery bypass grafting surgery: a prospective randomized clinical trial. Cardiol J.

[39] Wang H, Chen J, Zhao L (2018). N-3 polyunsaturated fatty acids for prevention of postoperative atrial fibrillation: updated meta-analysis and systematic review. J Interv Card Electrophysiol.

[40] Dvirnik N, Belley-Cote EP, Hanif H, Devereaux PJ, Lamy A, Dieleman JM, Vincent J, Whitlock RP (2018). Steroids in cardiac surgery: a systematic review and meta-analysis. Br J Anaesth.

[41] Imazio M, Brucato A, Ferrazzi P, Rovere ME, Gandino A, Cemin R, Ferrua S, Belli R, Maestroni S, Simon C (2011). Colchicine reduces postoperative atrial fibrillation: results of the Colchicine for the Prevention of the Postpericardiotomy Syndrome (COPPS) atrial fibrillation substudy. Circulation.

[42] Vizzardi E, D'Aloia A, Quinzani F, Bonadei I, Rovetta R, Bontempi L, Curnis A, Dei CL (2012). A focus on antiarrhythmic properties of ranolazine. J Cardiovasc Pharmacol Ther.

[43] Liu C, Wang J, Yiu D, Liu K (2014). The efficacy of glucocorticoids for the prevention of atrial fibrillation, or length of intensive care unite or hospital stay after cardiac surgery: a meta-analysis. Cardiovasc Ther.

[44] Auer J, Weber T, Berent R, Puschmann R, Hartl P, Ng CK, Schwarz C, Lehner E, Strasser U, Lassnig E (2004). A comparison between oral antiarrhythmic drugs in the prevention of atrial fibrillation after cardiac surgery: the pilot study of prevention of postoperative atrial fibrillation (SPPAF), a randomized, placebo-controlled trial. Am Heart J.

[45] Chen WT, Krishnan GM, Sood N, Kluger J, Coleman CI (2010). Effect of statins on atrial fibrillation after cardiac surgery: a duration- and dose-response meta-analysis. J Thorac Cardiovasc Surg.

[46] January CT, Wann LS, Alpert JS, Calkins H, Cigarroa JE, Cleveland JC, Conti JB, Ellinor PT, Ezekowitz MD, Field ME (2014). 2014 AHA/ACC/HRS guideline for the management of patients with atrial fibrillation: executive summary: a report of the American College of Cardiology/American Heart Association Task Force on practice guidelines and the Heart Rhythm Society. Circulation.

[47] Kirchhof P, Benussi S, Kotecha D, Ahlsson A, Atar D, Casadei B, Castella M, Diener HC, Heidbuchel H, Hendriks J (2016). 2016 ESC guidelines for the management of atrial fibrillation developed in collaboration with EACTS. Eur Heart J.

[48] Angelini GD, Penny WJ, El-Ghamary F, West RR, Butchart EG, Armistead SH, Breckenridge IM, Henderson AH (1987). The incidence and significance of early pericardial effusion after open heart surgery. Eur J Cardiothorac Surg.

[49] Goh SSC, Hamilton G, Stewart R, Thakur S, Murton M, Hardikar A (2016). Effect of posterior pericardiotomy on the incidence of postoperative atrial fibrillation in 1000 consecutive isolated CABG. Heart Lung Circulation.

[50] Sperling JS, Zapolanski AJ, Brizzio ME, Bronstein EH, Mindich BP (2009). Posterior pericardiotomy decreases the incidence and duration of atrial fibrillation after coronary artery bypass grafting. Innov Technol Tech Cardiothorac Vasc Surg.

[51] Gulmen S, Kiris I, Peker O, Yavuz T, Okutan H, Kuralay E, Ocal A (2010). The effect of posterior pericardiotomy on postoperative rhythm problems in coronary bypass surgery. Interact Cardiovasc Thorac Surg.

[52] Meza R, Gonzalez G, Franco G, Zapata J, Rendon JC, Montoya JD, Ramirez L, Fajardo D, Morales S, Jaramillo JS (2011). Effectiveness of posterior pericardiotomy in decreasing cardiac tamponade during the heart surgery postoperative period. Heart Surg Forum.

[53] Cakalagaoglu C, Koksal C, Baysal A, Alıcı G, Ozkan B, Boyacioglu K, Tasar M, Atasoy EB, Erdem H, Esen AM (2012). The use of posterior pericardiotomy technique to prevent postoperative pericardial effusion in cardiac surgery. Heart Surg Forum.

[54] Bakhshandeh AR, Salehi M, Radmehr H, Sattarzadeh R, Nasr AR, Sadeghpour AH (2009). Postoperative pericardial effusion and posterior pericardiotomy: related?. Asian Cardiovasc Thorac Ann.

