# Supplementary material for: Posterior pericardiotomy to prevent new-onset atrial fibrillation after coronary artery bypass grafting: a systematic review and meta-analysis of 10 randomized controlled trials
Source: J Cardiothorac Surg. 2021 Aug 14;16:233. doi: 10.1186/s13019-021-01611-x (PMC8364072; doi:10.1186/s13019-021-01611-x)
Supplement: Supplementary file 1 — Additional file 1: Appendix: Search strategy. [file 13019_2021_1611_MOESM1_ESM.pdf]

Search strategy for each database

Database	Search strategy
PubMed	("posterior pericardiotomy*[Title/Abstract]" OR "pericardial fenestration*[Title/Abstract]" OR "pericardialwindow*[Title/Abstract]") AND ("Coronary Artery Bypass"[Mesh]) OR ("Artery Bypass, Coronary*[Title/Abstract]" OR "Artery Bypasses, Coronary*[Title/Abstract]" OR "Bypasses, Coronary Artery*[Title/Abstract]" OR "Coronary Artery Bypasses*[Title/Abstract]" OR "Coronary Artery Bypass Surgery*[Title/Abstract]" OR "Bypass, Coronary Artery*[Title/Abstract]" OR "Aortocoronary Bypass*[Title/Abstract]" OR "Aortocoronary Bypasses*[Title/Abstract]" OR "Bypass, Aortocoronary*[Title/Abstract]" OR "Bypasses, Aortocoronary*[Title/Abstract]" OR "Bypass Surgery, Coronary Artery*[Title/Abstract]" OR "Coronary Artery Bypass Grafting*[Title/Abstract]") OR ("CAB*[Title/Abstract]" OR "CABG*[Title/Abstract]") OR("Cardiac Surgical Procedures"[Mesh]) OR ("Procedure, Cardiac Surgical*[Title/Abstract]" OR "Procedures, Cardiac Surgical*[Title/Abstract]" OR "Surgical Procedure, Cardiac*[Title/Abstract]" OR "Surgical Procedures, Cardiac*[Title/Abstract]" OR "Surgical Procedures, Heart*[Title/Abstract]" OR "Cardiac Surgical Procedure*[Title/Abstract]" OR "Heart Surgical Procedures*[Title/Abstract]" OR "Procedure, Heart Surgical*[Title/Abstract]" OR "Procedures, Heart Surgical*[Title/Abstract]" OR "Surgical Procedure, Heart*[Title/Abstract]" OR "Heart Surgical Procedure*[Title/Abstract]") OR "cardiothoracic surgery*[Title/Abstract]" OR "cardiac surgery*[Title/Abstract]" OR "heart surgery*[Title/Abstract]") OR ("Cardiopulmonary Bypass"[Mesh]) OR ("Heart-Lung Bypass*[Title/Abstract]" OR "Bypass, Heart-Lung*[Title/Abstract]" OR "Bypasses, Heart-Lung*[Title/Abstract]" OR "Heart Lung Bypass*[Title/Abstract]" OR "Heart-Lung Bypasses*[Title/Abstract]" OR "Bypass, Cardiopulmonary*[Title/Abstract]" OR "Bypasses, Cardiopulmonary*[Title/Abstract]" OR "Cardiopulmonary Bypasses*[Title/Abstract]") OR ("CPB*[Title/Abstract]")
Embase	('coronary artery bypass graft'/exp OR 'coronary artery bypass':ab,ti OR 'CAB':ab,ti OR 'heart surgery'/exp OR 'cardiac surgery':ab,ti OR 'cardiac surgical procedures':ab,ti OR 'cardiothoracic surgery':ab,ti OR 'cardiopulmonary bypass'/exp OR 'CBP':ab,ti) AND ('posterior pericardiotomy:ab,ti 'OR 'pericardial fenestration:ab,ti' OR 'pericardialwindow:ab,ti)
Cochrane Library	1. posterior pericardiotomy):ti,ab,kw OR (pericardial fenestration:ab,ti):ti,ab,kw OR (pericardialwindow:ab,ti):ti,ab,kw<br>2. CABG):ti,ab,kw OR (CAB):ti,ab,kw OR (cardiac surgery):ti,ab,kw OR (cardiothoracic surgery):ti,ab,kw OR (heart surgery):ti,ab,kw OR (CBP):ti,ab,kw<br>3. MeSH descriptor:[coronary atery bypass] explode all trees<br>4. Artery Bypass, Coronary):ti,ab,kw OR (Artery Bypasses, Coronary):ti,ab,kw OR (Bypasses, Coronary Artery):ti,ab,kw OR (Coronary Artery Bypasses):ti,ab,kw OR (Coronary Artery Bypass Surgery):ti,ab,kw OR (Bypass, Coronary Artery):ti,ab,kw

OR (Aortocoronary Bypass):ti,ab,kw OR (Aortocoronary Bypasses):ti,ab,kw OR (Bypass, Aortocoronary):ti,ab,kw OR (Bypasses, Aortocoronary):ti,ab,kw OR (Bypass Surgery, Coronary Artery):ti,ab,kw OR (Coronary Artery Bypass Grafting):ti,ab,kw

5. MeSH descriptor:[Cardiac Surgical Procedures] explode all trees
6. Procedure, Cardiac Surgical):ti,ab,kw OR (Procedures, Cardiac Surgical):ti,ab,kw OR (Surgical Procedure, Cardiac):ti,ab,kw OR (Surgical Procedures, Cardiac):ti,ab,kw OR (Surgical Procedures, Heart):ti,ab,kw OR (Cardiac Surgical Procedure):ti,ab,kw OR (Heart Surgical Procedures):ti,ab,kw OR (Procedure, Heart Surgical):ti,ab,kw OR (Procedures, Heart Surgical):ti,ab,kw OR (Surgical Procedure, Heart):ti,ab,kw OR (Heart Surgical Procedure):ti,ab,kw) OR (cardiothoracic surgery):ti,ab,kw OR (cardiac surgery):ti,ab,kw OR (heart surgery):ti,ab,kw
7. MeSH descriptor: [Cardiopulmonary Bypass] explode all trees
8. Heart-Lung Bypass):ti,ab,kw OR (Bypass, Heart-Lung):ti,ab,kw OR (Bypasses, Heart-Lung):ti,ab,kw OR (Heart Lung Bypass):ti,ab,kw OR (Heart-Lung Bypasses):ti,ab,kw OR (Bypass, Cardiopulmonary):ti,ab,kw OR (Bypasses, Cardiopulmonary):ti,ab,kwOR (Cardiopulmonary Bypasses):ti,ab,kw
9. #2 OR #3 OR #4 OR #5 OR #6 OR #7 OR #8
10. #1 AND #9
