# Supplementary material for: Posterior pericardiotomy to prevent new-onset atrial fibrillation after coronary artery bypass grafting: a systematic review and meta-analysis of 10 randomized controlled trials
Source: J Cardiothorac Surg. 2021 Aug 14;16:233. doi: 10.1186/s13019-021-01611-x (PMC8364072; doi:10.1186/s13019-021-01611-x)
Supplement: Supplementary file 2 — Additional file 2: Figure S1. Pooled analysis for the comparison of the risk for postoperative atrial fibrillation (POAF) after removal of the study with the smallest sample size. Figure S2. Pooled analysis for the comparison of the risk for postoperative atrial fibrillation (POAF) without preoperative oral β-blockers. Figure S3. Pooled analysis for the comparison of the risk for postoperative atrial fibrillation (POAF) in Turkey. Figure S4. Pooled analysis for the comparison of the risk for postoperative early pericardial effusion. Figure S5. Pooled analysis for the comparison of the risk for postoperative late pericardial effusion. Figure S6. Pooled analysis for the comparison of the risk for postoperative pericardial tamponade. Figure S7. Pooled analysis for the comparison of the risk for postoperative length of stay in intensive care unit (ICU). Figure S8. Pooled analysis for the comparison of the risk for postoperative pleural effusion. Figure S9. Pooled analysis for the comparison of the risk for postoperative length of hospitalization. Figure S10. Pooled analysis for the comparison of the risk for postoperative pulmonary complications. Figure S11. Pooled analysis for the comparison of the risk for postoperative revision for bleeding. Figure S12. Pooled analysis for the comparison of the risk for postoperative intra-aortic balloon pump (IABP) usage. Figure S13. Pooled analysis for the comparison of the risk for postoperative death. Table S1. Main postoperative data from random controlled trials included in the meta-analysis. Table S2. Actual mode of PP and use of posterior pericardial drains. [file 13019_2021_1611_MOESM2_ESM.pdf]

Figure S1. Pooled analysis for the comparison of the risk for postoperative atrial fibrillation (POAF) after removal of the study with the smallest sample size.

	PP		Control			Risk Ratio
Study or Subgroup	Events	Total	Events	Total	Weight	M-H, Random, 95% CI
Arbalti 2003	7	54	12	59	9.8%	0.64 [0.27, 1.50]
Asimakopoulos 1996	12	50	9	50	10.7%	1.33 [0.62, 2.88]
Ekim 2006	5	50	15	50	9.0%	0.33 [0.13, 0.85]
Farsak 2002	7	75	24	75	10.6%	0.29 [0.13, 0.64]
Fawzy 2015	13	100	30	100	12.9%	0.43 [0.24, 0.78]
Kaya 2014	6	30	11	33	9.7%	0.60 [0.25, 1.42]
Kaygin 2011	14	213	62	212	13.4%	0.22 [0.13, 0.39]
Kuralay 1999	6	100	34	100	10.2%	0.18 [0.08, 0.40]
Zhao 2013	20	228	35	230	13.7%	0.58 [0.34, 0.97]
**Total (95% CI)**		**900**		**909**	**100.0%**	**0.43 [0.29, 0.64]**
Total events	90		232			

Heterogeneity: Tau² = 0.23; Chi² = 22.35, df = 8 (P = 0.004); I² = 64%
Test for overall effect: Z = 4.19 (P < 0.0001)

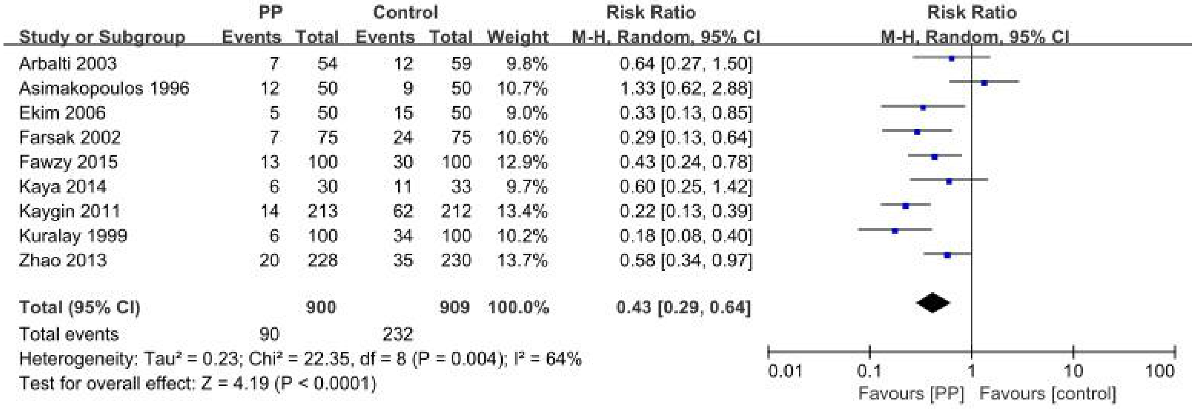

Figure S2. Pooled analysis for the comparison of the risk for postoperative atrial fibrillation (POAF) without preoperative oral β-blockers.

Study or Subgroup	PP Events	PP Total	Control Events	Control Total	Weight	Risk Ratio M-H, Fixed, 95% CI
Arbalti 2003	7	54	12	59	13.6%	0.64 [0.27, 1.50]
Ekim 2006	5	50	15	50	17.8%	0.33 [0.13, 0.85]
Farsak 2002	7	75	24	75	28.4%	0.29 [0.13, 0.64]
Kuralay 1999	6	100	34	100	40.3%	0.18 [0.08, 0.40]
**Total (95% CI)**		**279**		**284**	**100.0%**	**0.30 [0.20, 0.45]**
Total events	25		85			

Heterogeneity: $Chi^2 = 4.63$, df = 3 (P = 0.20); $I^2 = 35\%$

Test for overall effect: Z = 5.70 (P < 0.00001)

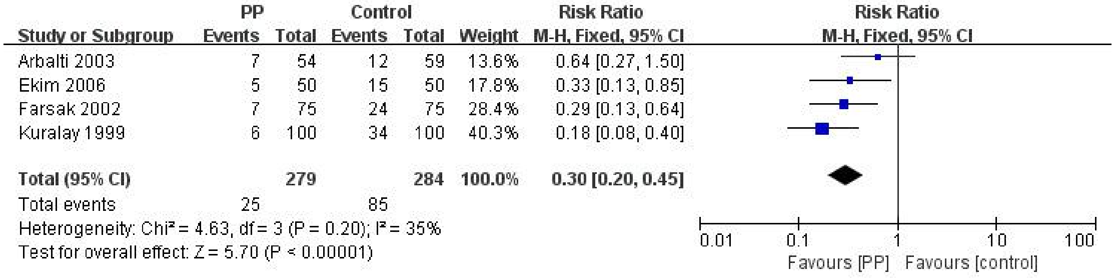

Figure S3. Pooled analysis for the comparison of the risk for postoperative atrial fibrillation (POAF) in Turkey.

	PP		Control			Risk Ratio
Study or Subgroup	Events	Total	Events	Total	Weight	M-H, Fixed, 95% CI
Arbalti 2003	0	0	0	0		Not estimable
Ekim 2006	7	54	12	59	9.3%	0.64 [0.27, 1.50]
Farsak 2002	5	50	15	50	12.2%	0.33 [0.13, 0.85]
Kaya 2014	7	75	24	75	19.5%	0.29 [0.13, 0.64]
Kaygin 2011	6	30	11	33	8.5%	0.60 [0.25, 1.42]
Kuralay 1999	14	213	62	212	50.5%	0.22 [0.13, 0.39]
**Total (95% CI)**		**422**		**429**	**100.0%**	**0.32 [0.23, 0.45]**
Total events	39		124			

Heterogeneity: Chi² = 6.17, df = 4 (P = 0.19); I² = 35%

Test for overall effect: Z = 6.69 (P < 0.00001)

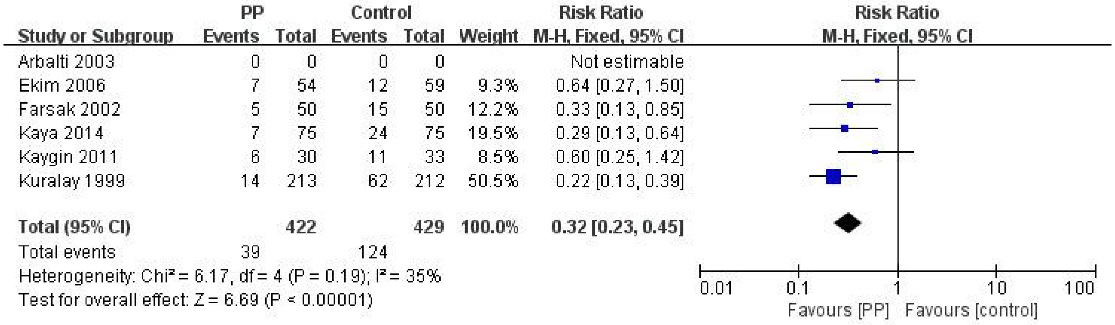

Figure S4. Pooled analysis for the comparison of the risk for postoperative early pericardial effusion.

	PP		Control			Risk Ratio
Study or Subgroup	Events	Total	Events	Total	Weight	M-H, Random, 95% CI
Arbalti 2003	14	54	28	59	14.1%	0.55 [0.32, 0.92]
Ekim 2006	6	50	21	50	12.2%	0.29 [0.13, 0.65]
Farsak 2002	8	75	32	75	13.0%	0.25 [0.12, 0.51]
Fawzy 2015	15	100	53	100	14.3%	0.28 [0.17, 0.47]
Kaya 2014	0	30	4	33	3.3%	0.12 [0.01, 2.17]
Kaygin 2011	10	213	46	212	13.3%	0.22 [0.11, 0.42]
Kongmalai 2014	7	10	6	10	13.3%	1.17 [0.61, 2.23]
Kuralay 1999	1	100	54	100	5.8%	0.02 [0.00, 0.13]
Zhao 2013	4	228	27	230	10.7%	0.15 [0.05, 0.42]
**Total (95% CI)**		**860**		**869**	**100.0%**	**0.28 [0.15, 0.50]**
Total events	65		271			

Heterogeneity: Tau² = 0.57; Chi² = 39.60, df = 8 (P < 0.00001); I² = 80%
Test for overall effect: Z = 4.26 (P < 0.0001)

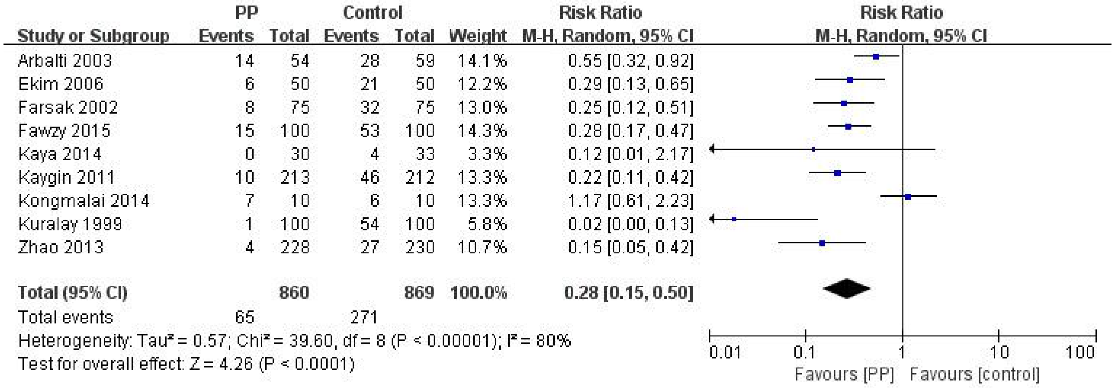

Figure S5. Pooled analysis for the comparison of the risk for postoperative late pericardial effusion.

Study or Subgroup	PP Events	PP Total	Control Events	Control Total	Weight	Risk Ratio M-H, Fixed, 95% CI
Ekim 2006	0	50	3	50	5.6%	0.14 [0.01, 2.70]
Farsak 2002	0	75	7	75	11.9%	0.07 [0.00, 1.15]
Kaya 2014	2	30	32	33	48.4%	0.07 [0.02, 0.26]
Kuralay 1999	0	100	21	100	34.1%	0.02 [0.00, 0.38]
**Total (95% CI)**		**255**		**258**	**100.0%**	**0.06 [0.02, 0.16]**
Total events	2		63			

Heterogeneity: Chi² = 0.86, df = 3 (P = 0.84); I² = 0%
Test for overall effect: Z = 5.41 (P < 0.00001)

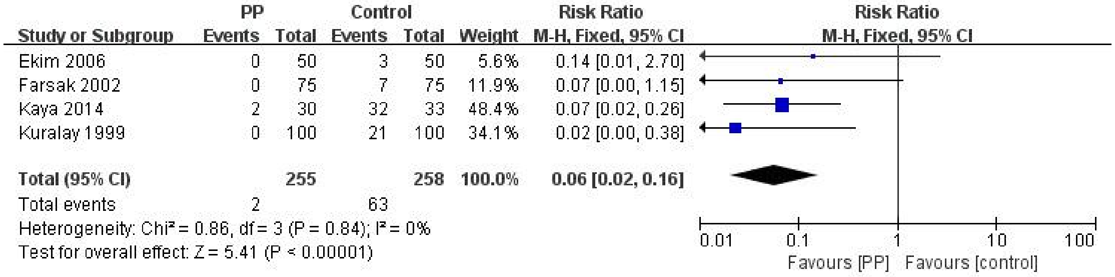

Figure S6. Pooled analysis for the comparison of the risk for postoperative pericardial tamponade.

	PP		Control			Risk Ratio
Study or Subgroup	Events	Total	Events	Total	Weight	M-H, Fixed, 95% CI
Farsak 2002	0	75	0	75		Not estimable
Fawzy 2015	0	100	3	100	13.6%	0.14 [0.01, 2.73]
Kaya 2014	0	30	4	33	16.6%	0.12 [0.01, 2.17]
Kongmalai 2014	0	213	7	212	29.1%	0.07 [0.00, 1.15]
Kuralay 1999	0	10	0	10		Not estimable
Zhao 2013	0	100	10	100	40.7%	0.05 [0.00, 0.80]
**Total (95% CI)**		**528**		**530**	**100.0%**	**0.08 [0.02, 0.33]**
Total events	0		24			

Heterogeneity: Chi² = 0.38, df = 3 (P = 0.94); I² = 0%
Test for overall effect: Z = 3.50 (P = 0.0005)

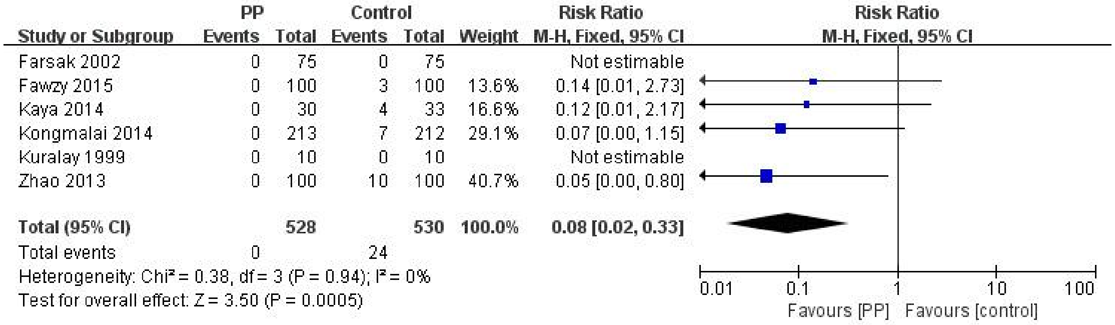

Figure S7. Pooled analysis for the comparison of the risk for postoperative length of stay in intensive care unit (ICU).

	PP			Control				Mean Difference
Study or Subgroup	Mean	SD	Total	Mean	SD	Total	Weight	IV, Fixed, 95% CI
Arbalti 2003	3	2	54	3	3	59	12.9%	0.00 [-0.93, 0.93]
Kongmalai 2014	4	2	10	2	2	10	3.6%	2.00 [0.25, 3.75]
Zhao 2013	3	2	228	2	2	230	83.5%	1.00 [0.63, 1.37]
**Total (95% CI)**			**292**			**299**	**100.0%**	**0.91 [0.57, 1.24]**

Heterogeneity: Chi² = 5.37, df = 2 (P = 0.07); I² = 63%
Test for overall effect: Z = 5.32 (P < 0.00001)

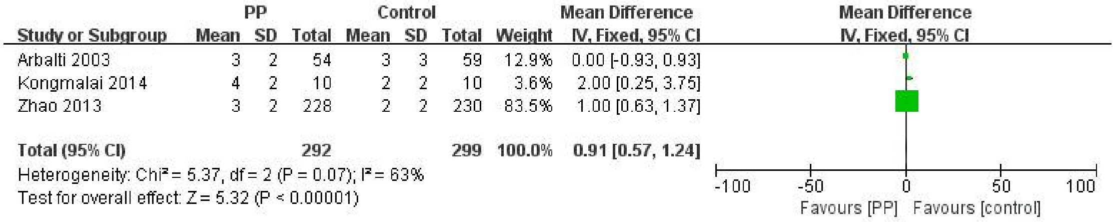

Figure S8. Pooled analysis for the comparison of the risk for postoperative pleural effusion.

	PP		Control			Risk Ratio
Study or Subgroup	Events	Total	Events	Total	Weight	M-H, Fixed, 95% CI
Arbalti 2003	7	54	3	59	3.4%	2.55 [0.69, 9.37]
Ekim 2006	12	50	9	50	10.8%	1.33 [0.62, 2.88]
Farsak 2002	19	75	13	75	15.6%	1.46 [0.78, 2.74]
Kongmalai 2014	10	10	5	10	6.6%	1.91 [1.04, 3.50]
Kuralay 1999	35	100	29	100	34.8%	1.21 [0.80, 1.81]
Zhao 2013	42	228	24	230	28.7%	1.77 [1.11, 2.82]
**Total (95% CI)**		**517**		**524**	**100.0%**	**1.51 [1.19, 1.92]**
Total events	125		83			

Heterogeneity: $Chi^2 = 2.91$, $df = 5$ ($P = 0.71$); $I^2 = 0\%$

Test for overall effect: $Z = 3.38$ ($P = 0.0007$)

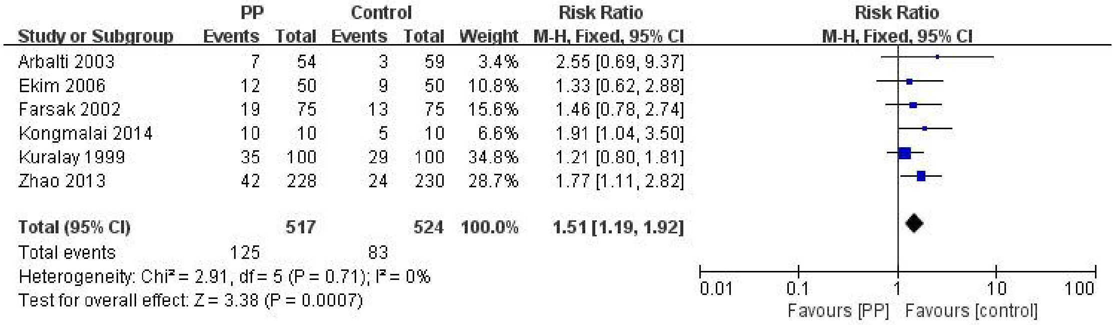

Figure S9. Pooled analysis for the comparison of the risk for postoperative length of hospitalization.

Study or Subgroup	PP Mean	PP SD	PP Total	Control Mean	Control SD	Control Total	Weight	Mean Difference IV, Fixed, 95% CI
Arbalti 2003	14	8	54	13	5	59	63.8%	1.00 [-1.49, 3.49]
Kaya 2014	7	3	30	12	11	33	25.9%	-5.00 [-8.90, -1.10]
Kongmalai 2014	16	6	10	14	8	10	10.3%	2.00 [-4.20, 8.20]
**Total (95% CI)**			**94**			**102**	**100.0%**	**-0.45 [-2.44, 1.54]**

Heterogeneity: Chi² = 7.13, df = 2 (P = 0.03); I² = 72%
Test for overall effect: Z = 0.44 (P = 0.66)

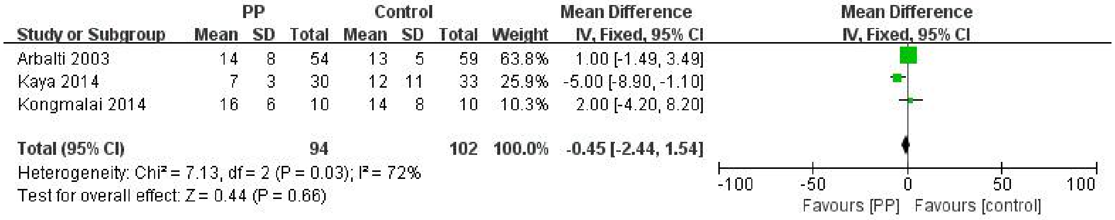

Figure S10.Pooled analysis for the comparison of the risk for postoperative pulmonary complications.

	PP		Control			Risk Ratio
Study or Subgroup	Events	Total	Events	Total	Weight	M-H, Fixed, 95% CI
Ekim 2006	2	50	3	50	5.3%	0.67 [0.12, 3.82]
Farsak 2002	3	75	2	75	3.5%	1.50 [0.26, 8.72]
Kaya 2014	4	30	12	33	20.0%	0.37 [0.13, 1.01]
Kaygin 2011	41	213	38	212	66.8%	1.07 [0.72, 1.60]
Kongmalai 2014	3	10	0	10	0.9%	7.00 [0.41, 120.16]
Kuralay 1999	3	100	2	100	3.5%	1.50 [0.26, 8.79]
**Total (95% CI)**		**478**		**480**	**100.0%**	**0.99 [0.71, 1.39]**
Total events	56		57			

Heterogeneity: Chi² = 6.26, df = 5 (P = 0.28); I² = 20%
Test for overall effect: Z = 0.04 (P = 0.97)

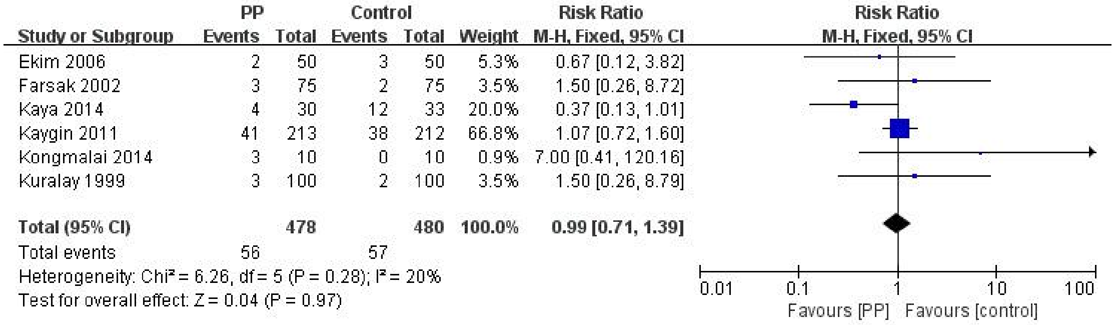

Figure S11. Pooled analysis for the comparison of the risk for postoperative revision for bleeding.

	PP		Control			Risk Ratio
Study or Subgroup	Events	Total	Events	Total	Weight	M-H, Fixed, 95% CI
Kaya 2014	0	30	2	33	13.3%	0.22 [0.01, 4.39]
Kaygin 2011	13	213	15	212	83.9%	0.86 [0.42, 1.77]
Kongmalai 2014	1	10	0	10	2.8%	3.00 [0.14, 65.90]
**Total (95% CI)**		**253**		**255**	**100.0%**	**0.84 [0.43, 1.63]**
Total events	14		17			

Heterogeneity: Chi² = 1.43, df = 2 (P = 0.49); I² = 0%
Test for overall effect: Z = 0.53 (P = 0.60)

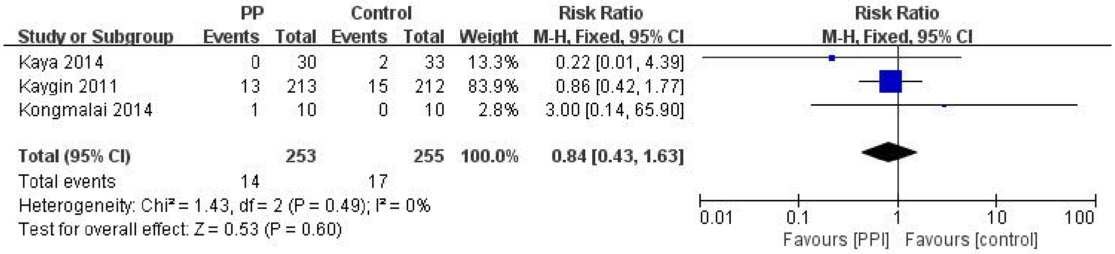

Figure S12. Pooled analysis for the comparison of the risk for postoperative intra aortic balloon pump (IABP) usage.

	PP		Control			Risk Ratio
Study or Subgroup	Events	Total	Events	Total	Weight	M-H, Fixed, 95% CI
Arbalti 2003	1	54	0	59	1.7%	3.27 [0.14, 78.67]
Asimakopoulos1996	1	50	1	50	3.6%	1.00 [0.06, 15.55]
Fawzy 2015	1	100	1	100	3.6%	1.00 [0.06, 15.77]
Kaygin 2011	24	213	25	212	91.0%	0.96 [0.56, 1.62]
Kongmalai 2014	0	10	0	10		Not estimable
**Total (95% CI)**		**427**		**431**	**100.0%**	**1.00 [0.61, 1.65]**
Total events	27		27			

Heterogeneity: Chi² = 0.56, df = 3 (P = 0.90); I² = 0%
Test for overall effect: Z = 0.00 (P = 1.00)

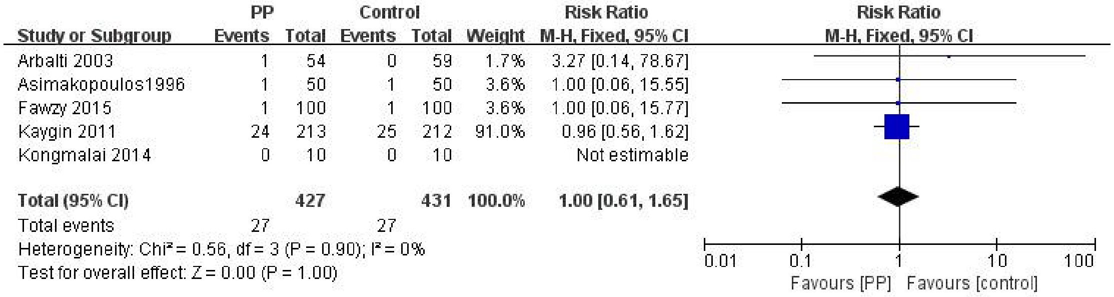

Figure S13. Pooled analysis for the comparison of the risk for postoperative death.

	PP		Control			Risk Ratio
**Study or Subgroup**	**Events**	**Total**	**Events**	**Total**	**Weight**	**M-H, Fixed, 95% CI**
Asimakopoulos 1996	1	50	1	50	29.5%	1.00 [0.06, 15.55]
Kaya 2014	0	30	2	33	70.5%	0.22 [0.01, 4.39]
**Total (95% CI)**		**80**		**83**	**100.0%**	**0.45 [0.07, 3.03]**
Total events	1		3			

Heterogeneity: Chi² = 0.55, df = 1 (P = 0.46); I² = 0%
Test for overall effect: Z = 0.82 (P = 0.41)

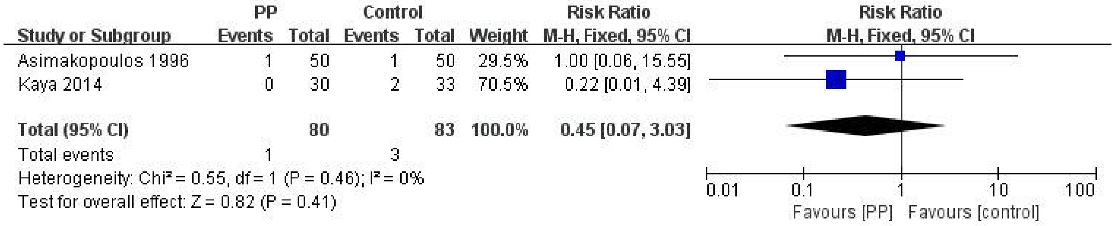

Table S1. Main postoperative data from random controlled trials included in the meta-analysis

StudyID	POAF		Early pericardial effusion		Late pericardial effusion		Pulmonary complication		Pericardial tamponade	
	PP	control	PP	control	PP	control	PP	control	PP	control
Kaya 2014[34]	6	11	0	4	2	32	4	12	0	4
Kaygin 2011[33]	14	62	10	46	NR	NR	41	38	0	7
Fawzy 2015[35]	13	30	15	53	NR	NR	NR	NR	0	3
Zhao 2013[29]	20	35	4	27	NR	NR	NR	NR	3	13
Kuralay 1999[18]	6	34	1	54	0	21	3	2	0	10
Asimakopoulos 1996[32]	12	9	NR	NR	NR	NR	NR	NR	NR	NR
Ekim 2006[20]	5	15	6	21	0	3	2	3	NR	NR
Farsak 2002[19]	7	24	8	32	0	7	3	2	0	0
Kongmalai 2014[22]	4	4	7	6	NR	NR	3	0	0	0
Arbalti 2003[21]	7	12	14	28	NR	NR	NR	NR	NR	NR

ICU stay，Days		Hospitalization time, days		IABP usage		Mortality		Revision for bleeding		Pleural effusion	
PP	control	PP	control	PP	control	PP	control	PP	control	PP	control
NR	NR	7±3	12±11	NR	NR	0	2	0	2	NR	NR
NR	NR	NR	NR	24	25	NR	NR	13	15	NR	NR
NR	NR	8	9	1	1	NR	NR	NR	NR	NR	NR
3±2	2±2	NR	NR	NR	NR	NR	NR	NR	NR	42	24
NR	NR	7	8	NR	NR	NR	NR	NR	NR	35	29
NR	NR	NR	NR	1	1	1	1	NR	NR	NR	NR
NR	NR	NR	NR	NR	NR	NR	NR	NR	NR	12	9
NR	NR	NR	NR	NR	NR	NR	NR	NR	NR	19	13
4±2	2±2	16±6	14±8	0	0	NR	NR	1	0	10	5
3±2	3±3	14±8	13±5	1	0	NR	NR	NR	NR	7	3

CABG,coronary artery bypass grafting; POAF,postoperative atrial fibrillation; ICU,intensive care unit; RCT,randomized controlled trial; NR, not reported; CPB,cardiopulmonary bypass; IABP,intra-aortic ballon pump.

Table S2. The actual mode of PP and the use of posterior pericardial drains

StudylD	the use of PP
Kaya 2014[34]	a 4-cm longitudinal incision was made parallel and posterior to the left phrenic nerve, extending from the left inferior pulmonary vein to the diaphragm
Kaygin2011[33]	a 4-cm longitudinal incision was made parallel and posterior to the left phrenic nerve, extending from the left inferior pulmonary vein to the diaphragm
Fawzy2015[35]	a longitudinal incision, 4-cm long and 2-cm width, was made parallel and posterior to the left phrenic nerve, extending from the left inferior pulmonary vein to the diaphragm
Zhao 2013[29]	The incision was made into the pleural cavity by noncontinuous electrocautery, parallel and posterior to the phrenic nerve and extending from the left inferior pulmonary vein to the diaphragm
Kuralay 1999[18]	Longitudinal incision was made parallel and posterior to the left phrenic nerve, extending from the left inferior pulmonary vein to the diaphragm
Asimakopoulos 1997[32]	a 4 cm posterior pericardial incision below the left inferior pulmonary vein parallel and posterior to the phrenic nerve
Ekim 2006[20]	A 4-cm longitudinal incision was made parallel and 1.5 cm posterior to the phrenic nerve, extending from the left inferior pulmonary vein to the diaphragm
Farsak2002[19]	4-cm longitudinal incision was made parallel and posterior to the left phrenic nerve, extending from the left inferior pulmonary vein to the diaphragm
Kongmalai 2014[22]	a 4-cm circular incision was made in parallel and posterior to the left phrenic nerve, extending from the left inferior pulmonary vein to the diaphragm
Arbalti 2003[21]	a longitudinal incision was made parallel and posterior to the phrenic nerve, extending from the left inferior pulmonary vein to the diaphragm

the use of posterior pericardial drains
A straight tube was placed in the anterior mediastinum and an angled tube was placed in the left hemithorax in all patients. The left pleural cavity was routinely opened
Two chest tubes (left pleural cavity and anterior mediastinum) were inserted into the pericardium.
Two chest tubes (one in the left pleural cavity and the second in the anterior mediastinum) were inserted
a 28 F rubber drain tube was placed along the right atrium in the pericardium and a 32 F silicone tube was placed retrosternally in the anterior mediastinum overlying the heart.The tubes were connected using a T-shaped,negative-pressure, drainage device.
Two chest tubes (one in the left pleural cavity and the other in anterior mediastinum) were inserted

Anterior mediastinal and left pleural drains were routinely placed with suction at 10 mm Hg

Two chest tubes were placed, one in the left pleural cavity and the other in the anterior mediastinum

Two chest tubes (one in the left pleural cavity and the other in anterior mediastinum)

One drainage tube was inserted into the left thorax and one or two into the anterior mediastinum, without suction

Two chest drains were inserted, one in the left pleural cavity and the other in the antermr mediastinum

---

PP,Posterior pericardiotomy
